# Post-GWAS functional analysis identifies CUX1 as a regulator of p16^INK4a^ and cellular senescence

**DOI:** 10.1038/s43587-022-00177-0

**Published:** 2022-02-17

**Authors:** Danli Jiang, Wei Sun, Ting Wu, Meijuan Zou, Sathish Babu Vasamsetti, Xiaoyu Zhang, Yihan Zhao, Julie A. Phillippi, Amr H. Sawalha, Sina Tavakoli, Partha Dutta, Jonathan Florentin, Stephen Y. Chan, Tammy S. Tollison, Di Wu, Jing Cui, Ian Huntress, Xinxia Peng, Toren Finkel, Gang Li

**Affiliations:** 1 Aging Institute, University of Pittsburgh, Pittsburgh, PA USA; 2 Center for Pulmonary Vascular Biology and Medicine, Pittsburgh Heart, Lung, Blood, and Vascular Medicine Institute, University of Pittsburgh School of Medicine and University of Pittsburgh Medical Center, Pittsburgh, PA USA; 3 Department of Medicine, Xiangya School of Medicine, Central South University, Changsha, China; 4 Department of Pharmacology, Nanjing Medical University, Nanjing, China; 5 Department of Cardiothoracic Surgery, University of Pittsburgh School of Medicine, Pittsburgh, PA USA; 6 Departments of Pediatrics Medicine, and Immunology & Lupus Center of Excellence, University of Pittsburgh School of Medicine, Pittsburgh, PA USA; 7 Departments of Radiology and Medicine, University of Pittsburgh, UPMC Presbyterian Hospital, Pittsburg, PA USA; 8 Department of Medicine, Division of Cardiology, University of Pittsburgh Medical Center, Pittsburgh, PA USA; 9 Department of Molecular Biomedical Sciences, North Carolina State University College of Veterinary Medicine, Raleigh, NC USA; 10 Department of Biostatistics, University of North Carolina at Chapel Hill, Chapel Hill, NC USA; 11 Division of Oral and Craniofacial Health Sciences, Adam School of Dentistry, University of North Carolina at Chapel Hill, Chapel Hill, NC USA; 12 Department of Medicine, Division of Rheumatology, Inflammation and Immunity, Brigham and Women’s Hospital, Boston, MA USA; 13 Bioinformatics Graduate Program, North Carolina State University, Raleigh, NC USA; 14 Bioinformatics Research Center, North Carolina State University, Raleigh, NC USA

**Keywords:** Senescence, Diseases, Ageing

## Abstract

Accumulation of senescent cells with age is an important driver of aging and age-related diseases. However, the mechanisms and signaling pathways that regulate senescence remain elusive. In this report, we performed post-genome-wide association studies (GWAS) functional studies on the *CDKN2A/B* locus, a locus known to be associated with multiple age-related diseases and overall human lifespan. We demonstrate that transcription factor CUX1 (Cut-Like Homeobox 1) specifically binds to an atherosclerosis-associated functional single-nucleotide polymorphism (fSNP) (rs1537371) within the locus and regulates the *CDKN2A/B-*encoded proteins p14^ARF^, p15^INK4b^ and p16^INK4a^ and the antisense noncoding RNA in the CDK4 (INK4) locus (ANRIL) in endothelial cells (ECs). Endothelial CUX1 expression correlates with telomeric length and is induced by both DNA-damaging agents and oxidative stress. Moreover, induction of CUX1 expression triggers both replicative and stress-induced senescence via activation of *p16*^*INK4a*^ expression. Thus, our studies identify CUX1 as a regulator of p16^INK4a^-dependent endothelial senescence and a potential therapeutic target for atherosclerosis and other age-related diseases.

## Abstract (web summary)

The molecular mechanisms that regulate senescence are incompletely understood. Here the authors couple high-throughput mapping of disease-associated functional SNPs (fSNPs) with proteomics analysis of fSNP-binding proteins to identify the transcription factor CUX1 as an activator of p16 expression and a regulator of senescence.

## Main

Since its discovery by three independent GWAS^1–3^, the *CDKN2A/B* locus on chromosome 9p21 has been noted as being strongly associated with the susceptibility of coronary artery disease^4^. This locus has also been associated with a multitude of additional cardiovascular conditions, including myocardial infarction and carotid artery plaque formation, in addition to other diseases such as type 2 diabetes and various forms of cancer^5,6^. However, GWAS cannot on their own specify the causative fSNPs associated with these diseases. Of note, all these diseases are recognized as age-related pathologies in that their incidence markedly increases with age^7–9^. Consistent with this observation, a genetic association of this locus with frailty and overall lifespan has also been recently revealed^10–12^. Since this single region associates with multiple age-related diseases, each with its own distinct pathophysiological mechanism, these data imply that this locus may contribute to the progression of these age-related diseases by modulating some aspect of aging biology as a common unifying mechanism^13^.

Aging is a continuous process of gradual functional decline^7^. Increasing evidence has implicated that the accumulation of senescent cells with age is a molecular driver of this functional decline, and also an important contributor to age-related diseases^9,14–20^. Cellular senescence is defined as irreversible cell cycle arrest often accompanied by an enlarged and flattened cellular morphology. In association with this arrest, senescent cells also secrete multiple proinflammatory molecules such as the cytokines IL-6 and IL-1β and the cell adhesion molecule ICAM1, collectively known as the senescence-associated secretory phenotype (SASP). These factors can induce both low-grade chronic inflammation and endothelial remodeling^21^. Based on the initiating trigger, cellular senescence can be classified as either replicative or stress induced. Although both types of senescence are mediated through the p53/p21 and/or p16^INK4a^/retinoblastoma protein (RB) pathways, preference for one pathway over the other depends on cell type, species and the stimuli^14,17,20,22^.

The *CDKN2A/B* locus, spanning a 200-kb region, harbors three well-characterized tumor suppressor genes—*p14*^*ARF*^*, p15*^*INK4b*^*, p16*^*INK4a*^—and ANRIL. Among these genes, *p16*^*INK4a*^ has been implicated in the *p16*^*INK4a*^/RB pathway that leads to cellular senescence in a variety of cell types^23–26^. In addition, *p16*^*INK4a*^ expression is also used as one of the common markers for cellular senescence^27^. Other senescence markers include senescence-associated β-galactosidase (SA-β-gal) staining, γ-H2AX, telomere length and expression of SASP genes^27^. However, relatively little is known about how *p16*^*INK4a*^ expression is regulated in response to various stimuli that trigger senescence.

In the present work, by coupling of regulatory element sequencing (Reel-seq) with flanking restriction-enhanced DNA pulldown–mass spectrometry (FREP–MS) and allele-imbalanced DNA pulldown–Western blot (AIDP–Wb), three techniques recently developed in our laboratory^28,29^, we discovered that CUX1, a transcription factor known to play roles in cell migration, proliferation, differentiation, DNA damage repair and tumorigenesis^30^, regulates both replicative and stress-induced senescence in human arterial ECs by modulation of *p16*^*INK4a*^ expression in response to telomere shortening, DNA damage and oxidative stress. We demonstrate that this regulation occurs via the specific binding of CUX1 to an atherosclerosis-associated fSNP, rs1537371, on the *CDKN2A/B* locus. Our findings reveal a new role of CUX1 in regulation of cellular senescence and provide new insights into how genetic variants in the *CDKN2A/B* locus can modulate susceptibility to atherosclerosis and other age-related complications.

## Results

### Identification of a fSNP rs1537371 within the *CDKN2A/B* locus

**Fig. 1:**
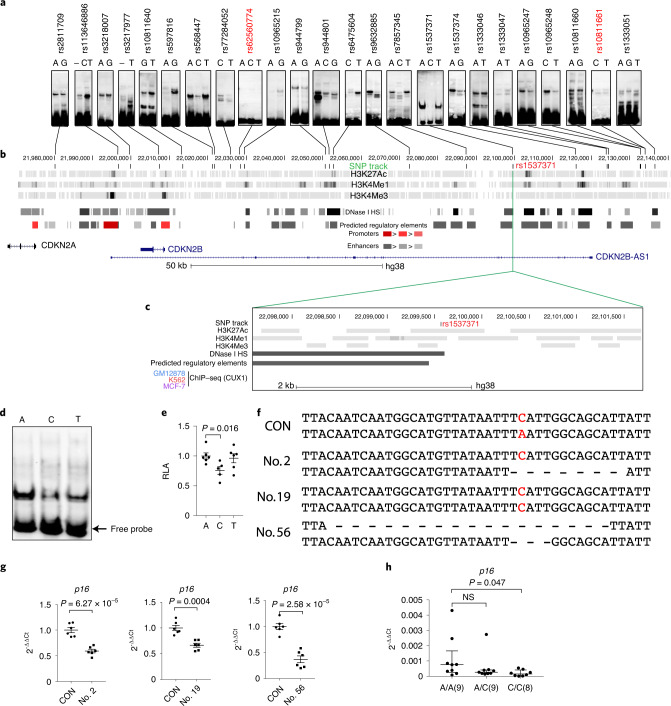
Identification and characterization of fSNP rs1537371. **a**, EMSA using NE isolated from human ECs, showing allele-imbalanced gel shifting on 22 of the 24 candidate fSNPs identified by Reel-seq screening of the *CDKN2A/B* locus using NE isolated from human PBMCs. Data for EMSA represent *n* = 3 biologically independent experiments. SNPs in red indicate no allele-imbalanced gel shifting. **b**, Genomic view of the 200-kb *CDKN2A/B* region showing the following tracks, ordered from top to bottom based on the ENCODE database. (1) SNP track showing locations of the 24 candidate fSNPs; (2–4) three epigenetic tracks for H3K27ac, H3K4me1 and H3K4me3, known as transcriptional factor-binding sites; (5) DNase I hypersensitivity sites (DNase I HS) in human astrocytes; (6) predicted regulatory elements including promoters (red) and enhancers (gray); (7) annotated genes including *p14*^*ARF*^, *p16*^*INK4a*^, *p15*^*INK4b*^ and ANRIL. **c**, Zoomed-in view of the 4-kb genomic region around fSNP rs1537371, showing the same tracks as above plus the negative result from CUX1 ChIP–seq assay in three human cell lines, GM12878, K562 and MCF-7. **d**,**e**, Demonstration of fSNP rs1537371 by EMSA (**d**) and luciferase reporter assay (**e**). A, risk allele; C, nonrisk allele; T, very rare allele; RLA, relative luciferase activity. Data for EMSA represent *n* = 3 biologically independent experiments; data for luciferase reporter assays represent *n* = 6 biologically independent samples. **f**, Sequences showing mutations around rs1537371 in three independent CRISPR–cas9 clones (nos. 2, 19 and 56), together with wild-type sequence. CON, wild-type control. **g**, qPCR showing decreased expression of *p16*^*INK4a*^, one of the potential risk genes in the three mutants. Data for qPCR analysis represent *n* = 3 biologically independent samples, each performed in duplicate. **h**, Dot plot of fSNP rs1537371 and *p16*^*INK4a*^ mRNA levels showing significantly higher expression of *p16*^*INK4a*^ in healthy PBMCs carrying homozygous risk allele A/A versus nonrisk allele C/C (*P* = 0.047, *n* = 26). *P* values were calculated using two-tailed Student’s *t*-test, and all data are presented as mean ± standard error (s.e.). **h**, Non-normally distributed data related to quantification of *p16*^*INK4a*^ expression are presented as median ± interquartile range, and *P* values were calculated with the nonparametric Mann–Whitney test for pairwise comparisons. Source data

Previously we demonstrated the feasibility of using Reel-seq to identify fSNPs associated with susceptibility to breast cancer^29^. By applying Reel-seq to the disease-associated *CDKN2A/B* locus, 24 candidate fSNPs were identified from 193 SNPs revealed by GWAS in linkage disequilibrium (LD), with *R*^2^ > 0.8 (Fig. 1a and Supplementary Data 1). The locations of these 24 candidate fSNPs are listed in the SNP track in Fig. 1b, indicting their overlap with transcription factor-binding sites, DNase I hypersensitivity sites or predicted promoters and enhancers^31^. Twenty-two of these candidate fSNPs were demonstrated as probable fSNPs using allele-imbalanced electrophoresis mobility shift assay (EMSA) as a criterion (Fig. 1a). Among these 22 fSNPs, SNP rs1537371 is in LD (*R*^2^ > 0.95) with a lead SNP, rs4977574, that is strongly associated with atherosclerosis in a European population^5^. To verify that rs1537371 is a fSNP, we first performed in silico analysis on this SNP using the Encyclopedia of DNA Elements (ENCODE) database. We found that rs1537371 is located in the H3K27ac (histone H3K27 acetylation), H3K4me1 (histone H3K4 methylation group 1) and DNase I hypersensitivity-enriched sites in both human umbilical vein endothelial cells (HUVECs) and human astrocytes (Fig. 1c). To further demonstrate that rs1537371 is functional, we repeated our EMSA analysis using nuclear extract (NE) isolated from primary ECs. This analysis revealed an allele-imbalanced gel shift pattern with the risk allele A noted to shift more than the nonrisk allele C (Fig. 1d). We next performed a luciferase reporter assay with both risk and nonrisk alleles in parallel. As noted with our EMSA results, allele-imbalanced luciferase reporter activity was also detected, with risk allele A having higher luciferase activity than nonrisk allele C (Fig. 1e). Finally, we performed CRISPR–cas9, a gene editing system with a synthetic guide RNA that targets the rs1537371 sequence in a human cell line. Three independent CRISPR-edited cell lines were generated (Fig. 1f). As indicated, clone 2 has a heterozygous deletion of 12 nucleotides, clone 56 has a 34-nucleotide deletion on one allele and a five-nucleotide deletion on the other while clone 19 carries a transversion from risk allele A to nonrisk allele C, which generates a homozygous C allele in this clone. Nevertheless, all these clones showed a significant reduction in *p16*^*INK4a*^ expression (Fig. 1g). In particular, downregulation of *p16*^*INK4a*^ in clone 19, carrying a C/C genotype compared to the C/A genotype of the parental control, suggests that risk allele A is a gain-of-function allele. To complement this CRISPR–cas9 analysis, we also examined *p16*^*INK4a*^ expression in peripheral blood mononuclear cells (PBMCs) of 26 unrelated individuals of varying genotype. We found that the nine individuals with homozygous risk allele A/A expressed a significantly higher level of *p16*^*INK4a*^ than the eight individuals carrying homozygous nonrisk allele C/C (*P* = 0.047, *n* = 17) as measured by quantitative PCR (qPCR; Fig. 1h). Together, these results support rs1537371 as a fSNP with allele-imbalanced activity.

### Identification of CUX1 specifically binding to rs1537371

To determine the protein(s) that specifically binds to rs1537371, we applied FREP–MS and identified six proteins binding it (Supplementary Table 1 and Supplementary Data 2). Among these six proteins we identified CUX1 as the top hit in this analysis. Of note, CUX1 is a member of the homeodomain family of DNA-binding proteins that have been reported to be involved in the regulation of cell proliferation, and to act as a tumor suppressor^30,32,33^.

**Fig. 2:**
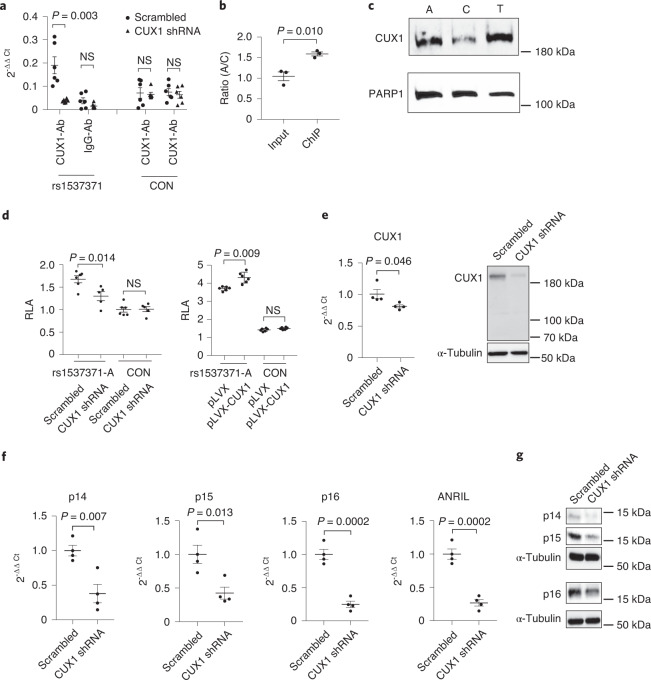
The role of CUX1 in regulation of *p14*^*ARF*^, *p15*^*INK4b*^, *p16*^*INK4a*^ and ANRIL expression via binding to fSNP rs1537371. **a**, ChIP assay demonstrating reduced binding of CUX1 to a DNA fragment containing rs1537371 in CUX1 shRNA knockdown ECs (left), and no specific binding of CUX1 to two randomly selected DNA fragments as controls (con; right). CUX1-Ab, anti-CUX1 antibody; IgG-Ab, anti-IgG antibody as an isotype control; NS, not significant. Data for ChIP assay represent *n* = 3 biologically independent experiments. **b**, Sequencing analysis showing significant enrichment of the A allele versus the C allele in ChIP DNA compared to input DNA (*n* = 3), with *P* = 0.010. **c**, AIDP–Wb demonstrating specific binding of CUX1 to rs1537371, with risk allele A binding more CUX1 than nonrisk allele C. T is a very rare allele. Data for AIDP–Wb represent *n* = 3 biologically independent experiments. **d**, CUX1-dependent luciferase reporter assay in 293T cells showing luciferase activity in *CUX1* shRNA knockdown (left) and CUX1-overexpressed ECs (right). pLVX-CUX1, CUX1 expression vector; rs1537371-A, luciferase reporter construct pGL3 (basic promoter vector, Promega); con, negative control. Data for this assay represent *n* = 6 biologically independent samples. **e**, qPCR (left) and immunoblot (right) showing downregulation of CUX1 in human ECs by shRNA knockdown. α-Tubulin was used as a loading control. Data for qPCR analysis represent *n* = 4 biologically independent samples, each performed in triplicate. Data for immunoblot analysis represent *n* = 3 biologically independent experiments. **f**, qPCR showing downregulation of *p14*^*ARF*^, *p15*^*INK4b*^, *p16*^*INK4a*^ and ANRIL expression in CUX1 shRNA knockdown human ECs. Data for qPCR analysis represent *n* = 4 biologically independent samples, each performed in triplicate. **g**, Immunoblot analysis showing downregulation of *p14*^*ARF*^, *p15*^*INK4b*^ and *p16*^*INK4a*^ expression in CUX1 shRNA knockdown human ECs. Data for immunoblot analysis represent *n* = 3 biologically independent experiments. *P* values were calculated using two-tailed Student’s *t*-test, and all data are presented as mean ± s.e.). Source data

To validate that CUX1 specifically binds to rs1537371, we first performed chromatin immunoprecipitation sequencing (ChIP–seq) using an anti-CUX1 antibody in ECs. We first demonstrated the specificity of this anti-CUX1 antibody by showing a significant enrichment of the rs1537371-containing DNA fragment pulled down through use of this antibody versus an anti-IgG antibody (Fig. 2a, left). Using this anti-CUX1 antibody, we observed a significant decrease in the binding of CUX1 to the rs1537371-containing DNA fragment in CUX1 short hairpin RNA (shRNA) knockdown ECs compared to scrambled shRNA control ECs (Fig. 2a, left). In contrast, when we performed the same ChIP assay on two randomly chosen genomic regions we did not observe any obvious difference in CUX1 binding (Fig. 2a, right). To confirm the allele-imbalanced binding of CUX1 to risk allele A versus nonrisk allele C, we performed Sanger sequencing since the ECs we used carry the A/C heterozygous genotype. After sequencing rs1537371-containing DNA fragments from both inputs and ChIP samples, we observed 55% enrichment of the A allele versus the C allele from the ChIP samples (24A/15C) compared to the inputs (20A/19C), with *P* < 0.01 (*n* = 3) (Fig. 2b). These data demonstrate an endogenous, allele-imbalanced binding of CUX1 to the fSNP rs1537371. Next, we performed AIDP–Wb, a new DNA pulldown assay recently developed in our laboratory, for specific detection of allele-imbalanced binding of a known protein to a given fSNP^29^. Using this technique, we were able to confirm the allele-imbalanced binding of CUX1 to the fSNP rs1537371, again with risk allele A binding more CUX1 than nonrisk allele C (Fig. 2c). The noted allele-imbalanced binding of CUX1 in this AIDP–Wb assay further validates the supposition that rs1537371 is indeed a bona fide fSNP. We next performed a luciferase reporter assay with a construct containing risk allele A used in Fig. 1e. We performed this assay in the setting of either CUX1 knockdown by shRNA or CUX1 overexpression by lentiviral expression vector pLVX. As expected, increased or decreased luciferase activity was observed when CUX1 expression was either positively or negatively modulated (Fig. 2d, left in both panels). This alteration in luciferase activity was not observed when we performed the same assay using a control reporter construct containing an irrelevant SNP sequence (Fig. 2d, right in both panels). These data suggest that CUX1 can bind to fSNP rs1537371 and regulate transcriptional activity. In addition, we also performed an online search and identified the core binding motif of CUX1 as ATC[A/C]AT^34^. This motif is highly similar to the sequence around rs1537371, AT[G/T]AAA. These data thus support our premise that rs1537371 is a fSNP and that CUX1 can bind to rs1537371 in an allele-imbalanced manner.

### *p14*^*ARF*^, *p15*^*INK4b*^, *p16*^*INK4a*^ and ANRIL are regulated by CUX1x

**Extended Data Fig. 1:**
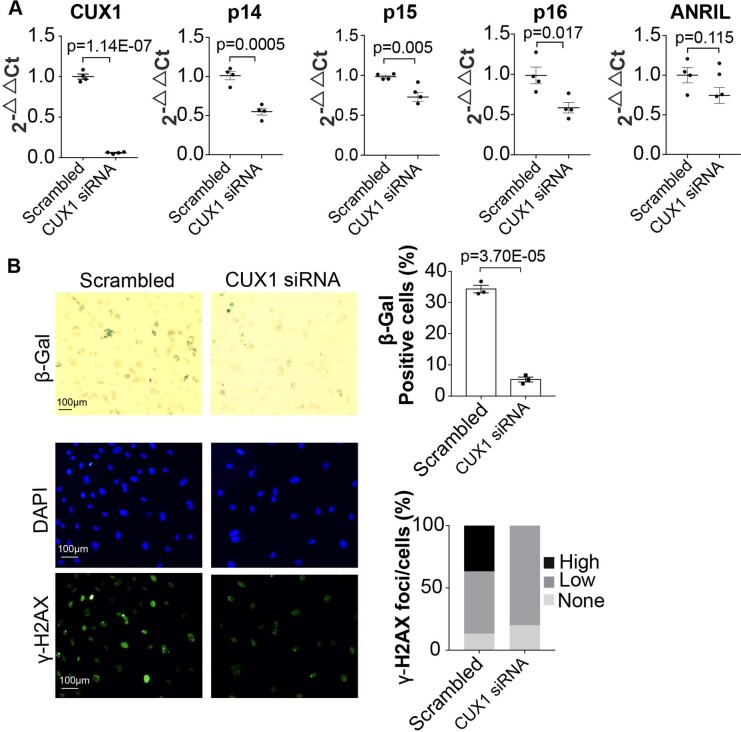
Characterization of the function of CUX1 by siRNA. **A**. qPCR analysis showing that a knockdown of *CUX1* by siRNA results in a downregulation of *p14*^*arf*^*, p15*^*INK4b*^*, p16*^*INK4a*^ and ANRIL expression in human primary ECs. Data for qPCR analysis represents four biologically independent samples (n=4), each performed in triplicate. **B**. A downregulation of CUX1 expression by siRNA in ECs represses replicative senescence (**right panel**) as is shown by both SA-β-gal (**upper**) and γ-H2AX (**lower**) staining. Data for SA-β-gal and γ-H2AX staining represents three biologically independent experiments (n=3). Quantitative plots for both β-gal positive cells (%) in SA-β-gal staining (**upper**) and γ-H2AX foci/cells (%) in γ-H2AX staining (**lower**) are shown on the right side of the panel. Source data

The 200-kb region of the *CDKN2A/B* locus contains three tumor suppressor genes—*p14*^*ARF*^, *p15*^*INK4b*^ *and p16*^*INK4a*^—as well as the long noncoding RNA, ANRIL. To determine whether these four genes are regulated by CUX1, we performed shRNA knockdown of CUX1 in primary ECs. Using a shRNA lentivirus that carries a sequence targeting the CUX1 gene, we were able to generate polyclonal pools of ECs with reduced expression of CUX1 as detected by both qPCR and immunoblot analysis (Fig. 2e). Of note, human ECs appeared to express only full-length CUX1 with an apparent molecular weight of 200 kDa and not the other proteolytic isoforms (p150, p110 and p75) as previously reported in various cancer cell lines^35,36^. Nevertheless, as a result of CUX1 downregulation, a significant decrease in the expression of *p14*^*ARF*^, *p15*^*INK4b*^, *p16*^*INK4a*^ and ANRIL was evidenced at the messenger RNA level (Fig. 2f) and, in addition, decreased expression of coding proteins p14^ARF^, p15^INK4b^ and p16^INK4a^ was detected by immunoblot analysis (Fig. 2g). These results thus demonstrate that CUX1 can modulate the expression of *p14*^*ARF*^, *p15*^*INK4b*^, *p16*^*INK4a*^ and ANRIL. To confirm these results, we also performed the same knockdown in ECs using a small interfering RNA (siRNA) that targets a CUX1 sequence different from that employed in CUX1 shRNA knockdown. This siRNA approach also resulted in a significant reduction in the expression of all four genes within the *CDKN2A/B* locus (Extended Data Fig. 1a). These data, together with data generated by various independent strategies (for example, CRISPR–cas9 gene editing, ChIP, AIDP-Wb, CUX1-dependent luciferase reporter assay and in silico analysis), support the view that CUX1, as a transcription factor, regulates the expression of risk genes *p14*^*ARF*^, *p15*^*INK4b*^, *p16*^*INK4a*^ and ANRIL by binding to the fSNP rs1537371.

### CUX1 regulates replicative senescence via p16^INK4a^

**Fig. 3:**
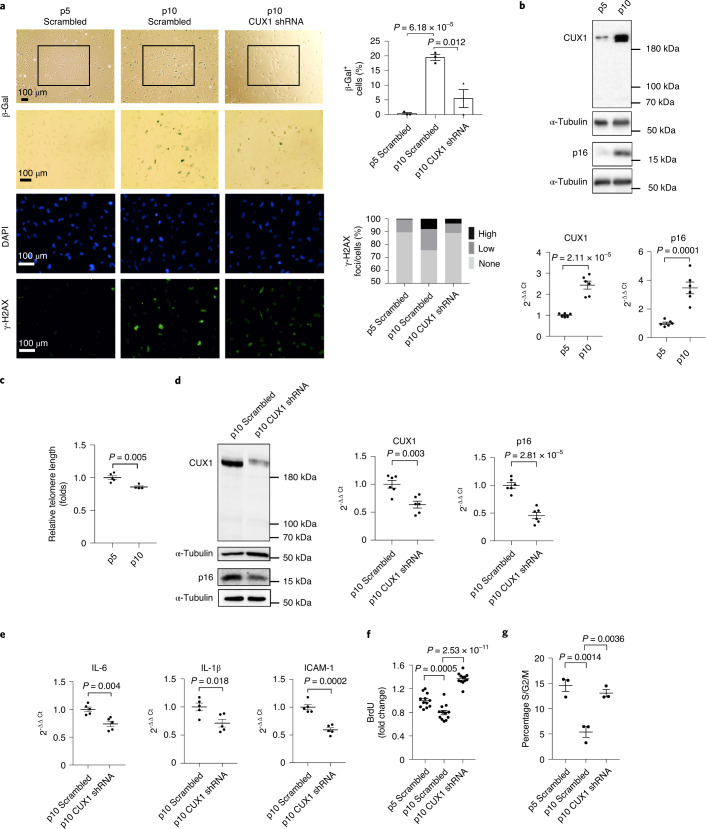
CUX1 regulates replicative senescence in ECs. **a**, SA-β-gal (top) and γ-H2AX staining (bottom) showing an increase in replicative senescence from p5 ECs (left) to p10 ECs (middle), and a reduction in replicative senescence in CUX1 shRNA knockdown p10 ECs (right) compared to scrambled p10 ECs (middle). Right, quantitative plots are shown for both β-gal^+^ cells (%) in SA-β-gal staining (top) and γ-H2AX foci/cells (%) with γ-H2AX staining (bottom). Data for SA-β-gal and γ-H2AX staining represent *n* = 3 biologically independent experiments. **b**, Immunoblots and qPCR showing increased expression of *CUX1* and *p16*^*INK4a*^ in p10 ECs compared to p5 ECs. Data for immunoblot analysis represent *n* = 3 biologically independent experiments; data for qPCR analysis represent *n* = 3 biologically independent samples, each performed in duplicate. **c**, PCR-based analysis showing significant decrease in telomeric length from p5 to p10 ECs. Data for PCR analysis represent *n* = 4 biologically independent samples. **d**, Immunoblots and qPCR showing that shRNA knockdown of CUX1 resulted in decreased expression of *p16*^*INK4a*^ in p10 human ECs. Data for immunoblot analysis represent *n* = 3 biologically independent experiments; data for qPCR analysis represent *n* = 3 biologically independent samples, each performed in duplicate. **e**, qPCR showing significant downregulation of SASP genes, IL-6, IL-1β and ICAM1 in CUX1 shRNA knockdown p10 ECs. Data for qPCR analysis represent *n* = 3 biologically independent samples, each performed in duplicate. **f**,**g**, Decrease in both BrdU incorporation (**f**) and percentage of S/G2/M cell numbers (**g**) in p10 ECs (middle) versus p5 ECs (left) indicated an increase in replicative senescence. Knockdown of CUX1 in p10 ECs (right) resulted in recovery from both decreased BrdU incorporation and reduced percentage of S/G2/M cell numbers, indicating a blockage in cellular senescence in CUX1 shRNA knockdown p10 ECs. Data for BrdU incorporation represent *n* = 12 biologically independent samples; data for cell cycle analysis represent *n* = 3 biologically independent samples. *P* values were calculated using two-tailed Student’s *t*-test, and all data are presented as mean ± s.e.). Source data

Because *p16*^*INK4a*^ has been implicated in senescence^9,14,37^, we reasoned that CUX1-mediated regulation of *p16*^*INK4a*^ expression might have important implications in endothelial senescence. To test this hypothesis, we first performed both SA-β-gal and γ-H2AX staining of primary human ECs collected at either early passage (p5) or late passage (p10). This analysis revealed the expected increase in both SA-β-gal^+^ cells (Fig. 3a, top) and γ-H2AX foci (Fig. 3a, bottom) in p10 ECs (Fig. 3a, middle) versus p5 ECs (Fig. 3a, left). We next sought to determine correlation between the expression of *CUX1* and *p16*^*INK4a*^. As a result, passage-dependent increase in *CUX1* expression and a concomitant increase in that of p16^INK4a^ from p5 to p10 ECs were detected at both mRNA and protein levels (Fig. 3b). In addition, using a PCR-based assay, we also detected decreased telomere length in p10 versus p5 ECs (Fig. 3c), suggesting a negative correlation between the expression level of CUX1 and telomere length. Together, these data support a potential role of CUX1 in regulation of replicative senescence.

To demonstrate that CUX1 is responsible for replicative senescence in ECs, we infected p10 ECs with a lentiviral shRNA targeting CUX1. Forty-eight hours after infection, downregulation of CUX1 and decreased expression of *p16*^*INK4a*^ were confirmed by both immunoblot and qPCR analysis (Fig. 3d). In these CUX1 shRNA knockdown p10 ECs, a significant decrease in both SA-β-gal and γ-H2AX staining was detected (Fig. 3a, right) in comparison with the scrambled control (Fig. 3a, middle), suggesting a reversal of cellular senescence in these cells. In addition, we also detected a significant downregulation of various SASP genes, including *IL-6*, *IL-1β* and *ICAM1*, in CUX1 shRNA knockdown p10 ECs (Fig. 3e). These data demonstrate that CUX1 can modulate replicative senescence. Similar findings were also observed when CUX1 expression was modulated independently by siRNA knockdown in ECs (Extended Data Fig. 1b). Moreover, because cell cycle arrest is an essential feature of cellular senescence^38^, we also performed a 5-bromo-2’-deoxyuridine (BrdU) incorporation assay and cell cycle analysis. As shown in Fig. 3f,g, a significant decrease in both BrdU incorporation and the percentage of cell numbers in the S/G2/M phase was observed in p10 ECs (right) versus p5 ECs (left), suggesting potential cell cycle arrest. Consistent with the recovered SA-β-gal and γ-H2AX staining in CUX1 shRNA knockdown p10 ECs (Fig. 3a), reduction in both BrdU incorporation and percentage of cell numbers in the S/G2/M phase can also be reversed by CUX1 shRNA knockdown in p10 ECs (Fig. 3f,g, right).

**Extended Data Fig. 2:**
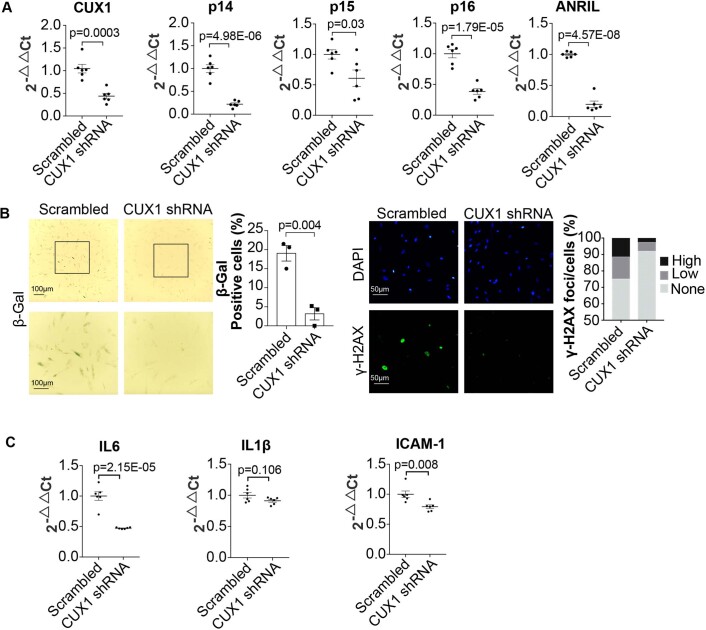
Characterization of the CUX1 function in human VSMCs. **A**. qPCR analysis showing *CUX1* knockdown by shRNA results in a downregulation of *p16*^*INK4a*^*, p14*^*ARF*^*, p15*^*INK4b*^ and ANRIL expression in human primary VSMCs. Data for qPCR represents three biologically independent samples (n=3), each performed in duplicate. **B**. SA-β-gal (**left**) and γ-H2AX (**right**) staining showing that a knockdown of CUX1 in human primary VSMCs inhibits replicative senescence (**right panel**)(n=3). Quantitative plots for both β-gal positive cells (%) in SA-β-gal staining and γ-H2AX foci/cells (%) in γ-H2AX staining are shown on the right side of the panel. **C**. qPCR analysis showing a significant decrease in the expression of *IL-6* and *ICAM1*, but not *IL1β* in the CUX1 shRNA knockdown VSMCs (**right lane**). Data for qPCR analysis represents three biologically independent samples (n=3), each performed in duplicate. Source data

In addition, to determine whether the regulation of replicative senescence by CUX1 is specific for ECs, we performed CUX1 shRNA knockdown in human primary vascular smooth muscle cells (VSMCs). Consistent with the results we observed in ECs, downregulation of CUX1 in VSMCs also resulted in a significant decrease in the expression of *p14*^*ARF*^, *p15*^*INK4b*^, *p16*^*INK4a*^ and ANRIL (Extended Data Fig. 2a). This was accompanied by a significant decrease in both SA-β-gal and γ-H2AX staining (Extended Data Fig. 2b), as well as reduced expression of SASP genes (Extended Data Fig. 2c). These data indicate that the role of CUX1 in regulation of replicative senescence is unlikely to be EC specific: it could also occur in other cell types relevant to atherogenesis, such as VSMCs.

### Activation of cellular senescence by CUX1 requires p16^INK4a^

**Fig. 4:**
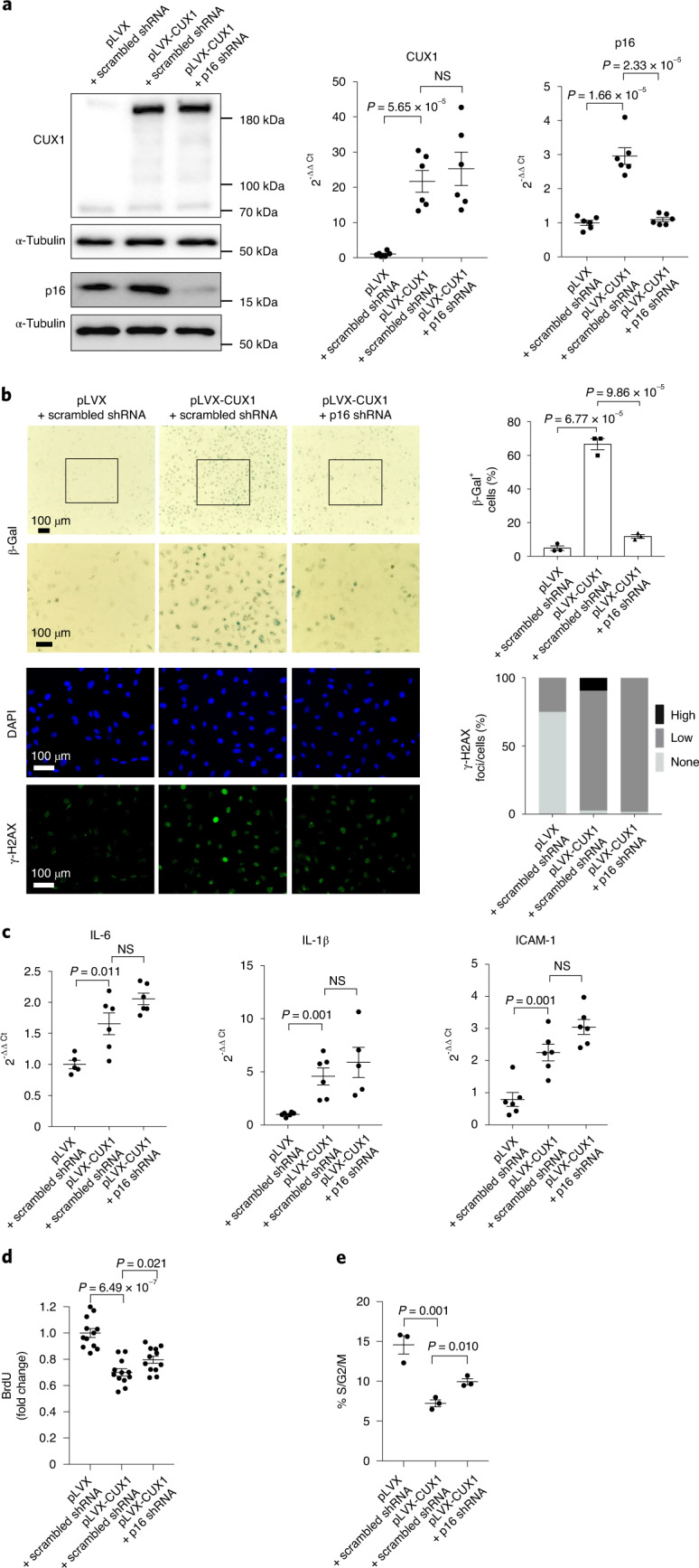
p16^INK4a^ is the downstream mediator of CUX1-regulated senescence. **a**, Immunoblot and qPCR analysis demonstrating that overexpression of CUX1 results in increased expression of p16^INK4a^ (middle) in ECs. Increased *p16*^*INK4a*^ expression was repressed by *p16*^*INK4a*^ shRNA knockdown (right). Data for immunoblot analysis represent *n* = 3 biologically independent experiments; data for qPCR analysis represent *n* = 3 biologically independent samples, each performed in duplicate. **b**, SA-β-gal (top) and γ-H2AX (bottom) staining showing that overexpression of CUX1-induced cellular senescence in human ECs (middle versus left). Downregulation of *p16*^*INK4a*^ by shRNA in CUX1-overexpressed human ECs rescued senescent phenotypes (right; *n* = 3). Quantitative plots for both β-gal^+^ cells (%) in SA-β-gal staining and γ-H2AX foci/cells (%) in γ-H2AX staining are shown. **c**, qPCR showing increased expression of SASP genes *IL-6*, *IL-1β* and *ICAM1* in CUX1-overexpressed ECs (middle versus left). Increased expression of SASP genes IL-6, IL-1β and ICAM1 remained unchanged in CUX1-overexpressed and *p16*^*INK4a*^ shRNA downregulated human ECs (right). Data for SA-β-gal and γ-H2AX staining represent *n* = 3 biologically independent experiments; data for qPCR analysis represent *n* = 3 biologically independent samples, each performed in duplicate. **d**,**e**, Decrease in BrdU incorporation (**d**) and percentage of S/G2/M cell numbers (**e**) in CUX1-overexpressed human ECs (middle) demonstrated an increase in cellular senescence. Knockdown of *p16*^*INK4a*^ by shRNA in CUX1-overexpressed human ECs (right) resulted in recovery from decreased BrdU incorporation and reduced percentage of S/G2/M cell numbers, indicating blockage of cellular senescence in CUX1-overexpressed and *p16*^*INK4a*^-downregulated human ECs. Data for BrdU incorporation represent *n* = 12 biologically independent samples; data for cell cycle analysis represent *n* = 3 biologically independent samples. *P* values were calculated using two-tailed Student’s *t*-test, and all data are presented as mean ± s.e. Source data

Among the four genes regulated by CUX1, *p16*^*INK4a*^ is known as a regulator of cellular senescence^9,14^. To determine whether *p16*^*INK4a*^ is the downstream mediator of CUX1 responsible for regulation of cellular senescence, we first overexpressed CUX1 in human ECs using the above-mentioned lentiviral expression vector pLVX-CUX1. An upregulation of p16^INK4a^ induced by CUX1 overexpression was demonstrated at both the protein and mRNA level as detected by immunoblot and qPCR analysis, respectively (Fig. 4a, left and middle). Consistent with the increased expression of *p16*^*INK4a*^, CUX1-overexpressed human ECs showed an increased level of cellular senescence as demonstrated by enhanced staining of both SA-β-gal and γ-H2AX (Fig. 4b, left and middle), increased expression of various SASP genes including *IL-6*, *IL-1β* and *ICAM1* (Fig. 4c, left and middle) as well as a reduction in both BrdU incorporation (Fig. 4d, left and middle) and the percentage of cell numbers in the S/G2/M phase (Fig. 4e, left and middle). To further demonstrate that CUX1 activates cellular senescence by upregulation of *p16*^*INK4a*^, we downregulated *p16*^*INK4a*^ expression using a shRNA lentivirus in CUX1-overexpressed human ECs. As can be seen in Fig. 4a (middle and right), reduction in p16^INK4a^ expression was evidenced at both the protein and mRNA level. Consistently, knockdown of *p16*^*INK4a*^ in CUX1-overexpressed cells mitigated an increase in senescence, as demonstrated by a decrease in both SA-β-gal and γ-H2AX staining (Fig. 4b, middle and right) as well as an increase in both BrdU incorporation (Fig. 4d, middle and right) and the percentage of cell numbers in the S/G2/M phase (Fig. 4e, middle and right). Together, these data suggest that CUX1 induces senescence in a *p16*^*INK4a*^-independent manner. However, the increased expression level of SASP genes *IL-6*, *IL-1β* and *ICAM1* in CUX1-overexpressed ECs remained unchanged following *p16*^*INK4a*^ knockdown (Fig. 4c, right). These data are consistent with a previous publication showing that *p16*^*INK4a*^ can induce cellular senescence without the associated inflammatory secretory phenotypes^39^.

**Extended Data Fig. 3:**
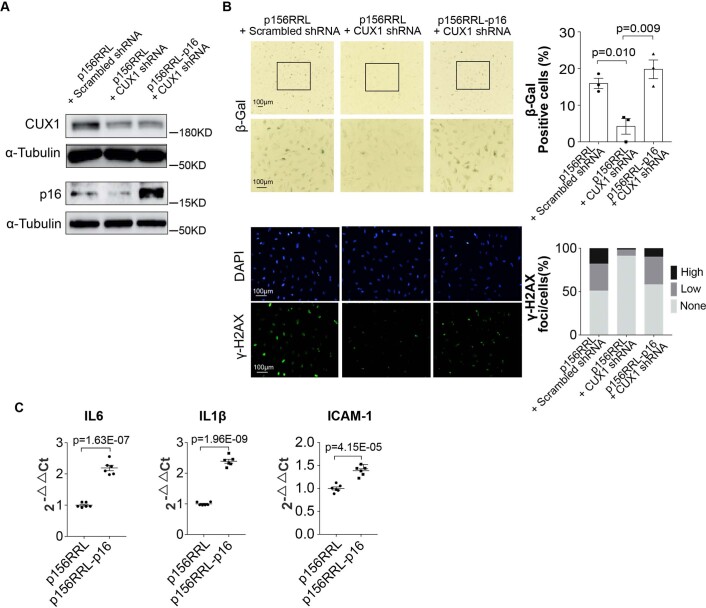
A functional complementation analysis demonstrating that CUX1 regulates replicative senescence by activating the p16^INK4a^ expression. **A**. Western blot analysis showing an over-expression of p16^INK4a^ using an ectopic vector p156RRL in the CUX1 shRNA knockdown ECs (**right lane**). A knockdown of CUX1 resulted in a downregulation of p16^INK4a^ (**middle lane**). Data for the Western blot analysis represents three biologically independent experiments (n=3). **B**. SA-β-gal (**upper**) and γ-H2AX (**lower**) staining showing the restoration of replicative senescence in the CUX1 shRNA knockdown and p16^INK4a^ over-expressed ECs (**right panel**). Quantitative plots for both β-gal positive cells (%) in SA-β-gal staining and γ-H2AX foci/cells (%) in γ-H2AX staining are shown on the right side of the panel. Data for SA-β-gal and γ-H2AX staining represents three biologically independent experiments (n=3). **C**. qPCR analysis of the SASP genes *IL-6*, *IL1β* and *ICAM1* showing that an over-expression of p16^INK4a^ in the CUX1 shRNA knockdown human ECs rescued the expression of *IL-6*, *IL1β* and *ICAM1* (**right lane**). Data for qPCR analysis represents three biologically independent samples (n=3), each performed in duplicate. Source data

To further demonstrate that *p16*^*INK4a*^ is the downstream mediator of CUX1-induced cellular senescence, we also overexpressed *p16*^*INK4a*^ in CUX1 shRNA knockdown ECs. As expected, knockdown of CUX1 resulted in decreased expression of p16^INK4a^ in human ECs (Extended Data Fig. 3a, middle). Under this condition, reduced cellular senescence was observed as demonstrated by a decrease in both SA-β-gal and γ-H2AX staining (Extended Data Fig. 3b, middle). Consistent with this observation, when we overexpressed p16^INK4a^ using lentiviral expression vector p156RRL in CUX1 shRNA knockdown ECs (Extended Data Fig. 3a, right), we detected restoration of both SA-β-gal and γ-H2AX staining (Extended Data Fig. 3b, right), as well as in the expression of SASP genes *IL-6*, *IL-1β* and *ICAM1* (Extended Data Fig. 3c, right). Of note, restoration of the expression of these SASP factors in *p16*^*INK4a*^-overexpressed ECs was unexpected since, as mentioned above, *p16*^*INK4a*^ is not believed to be a SASP-inducing factor^39^.

**Extended Data Fig. 4:**
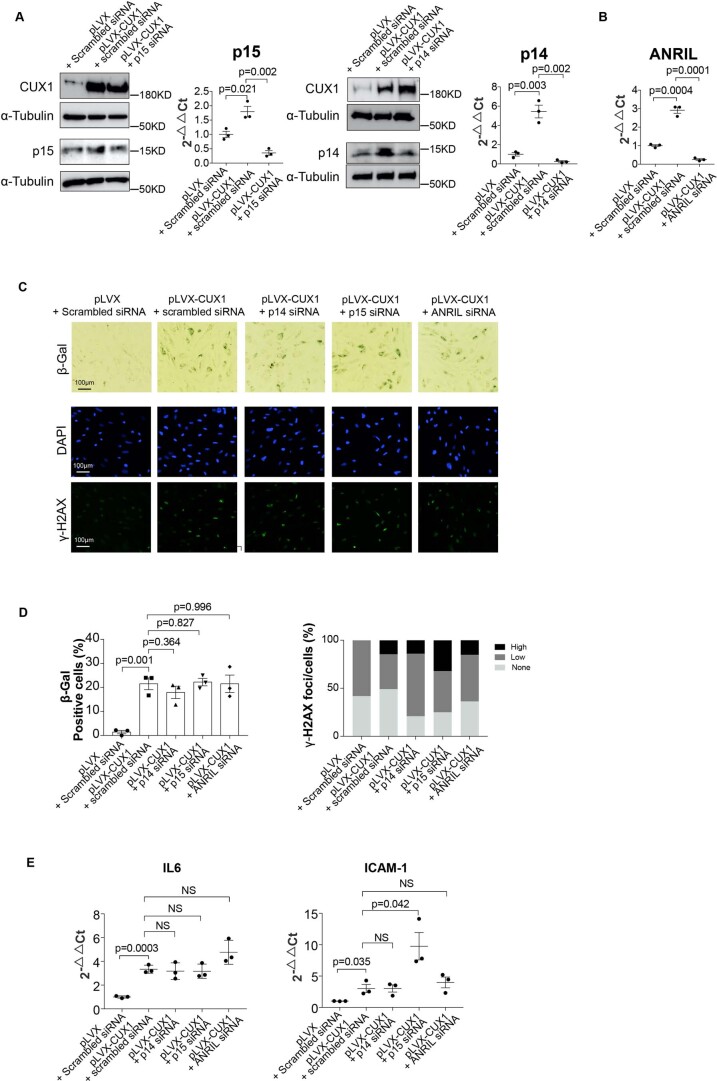
A functional analysis assessing the role of *p14*^*ARF*^*, p15*^*INK4b*^ and ANRIL in regulating cellular senescence. **A**. Western blot analysis and qPCR showing an over-expression of CUX1 in human ECs (**middle lane**) resulted in an upregulation of *p14*^*ARF*^ and *p15*^*INK4b*^. The upregulation of *p14*^*ARF*^*, and p15*^*INK4b*^ can be blocked by RNAi knockdown (**right lane**). Data for the Western blot analysis represents three biologically independent experiments (n=3). Data for qPCR analysis represents three biologically independent experiments (n=3). **B**. qPCR showing an over-expression of CUX1 in human ECs (**middle lane**) resulted in an upregulation of ANRIL. The upregulation of ANRIL can be inhibited by RNAi knockdown (**right lane**). Data for qPCR analysis represents three biologically independent experiments (n=3). **C**. SA-β-gal (**upper**) and γ-H2AX (**lower**) staining demonstrating that the increase in cellular senescence in the CUX1 over-expressed human ECs cannot be inhibited by the downregulation of *p14*^*ARF*^*, p15*^*INK4b*^ or ANRIL. **D**. Quantitative plots for both β-gal positive cells (%) in SA-β-gal staining and γ-H2AX foci/cells (%) in γ-H2AX staining are shown on the right side of the panel. Data for SA-β-gal and γ-H2AX staining represents three biologically independent experiments (n=3). **E**. qPCR analysis of the SASP genes *IL-6* and *ICAM1* showing that a knockdown of *p14*^*ARF*^*, p15*^*INK4b*^, and ANRIL did not decrease the CUX1-induced expression of SASP genes. Data for qPCR analysis represents three biologically independent samples (n=3), each performed in duplicate. pLVX: lentiviral vector for gene expression. Source data

Since CUX1 also regulates the expression of *p14*^*ARF*^*, p15*^*INK4b*^ and ANRIL (Fig. 2f,g), we next sought to determine whether these gene products play a role in regulation of endothelial senescence. We therefore performed siRNA knockdown of *p14*^*ARF*^, *p15*^*INK4b*^ and ANRIL in CUX1-overexpressed human ECs (Extended Data Fig. 4a,b). However, we noted no obvious change in endothelial senescence following knockdown of *p14*^*ARF*^, *p15*^*INK4b*^ or ANRIL (Extended Data Fig. 4c–e).

### CUX1 regulates stress-induced senescence via p16^INK4a^

**Fig. 5:**
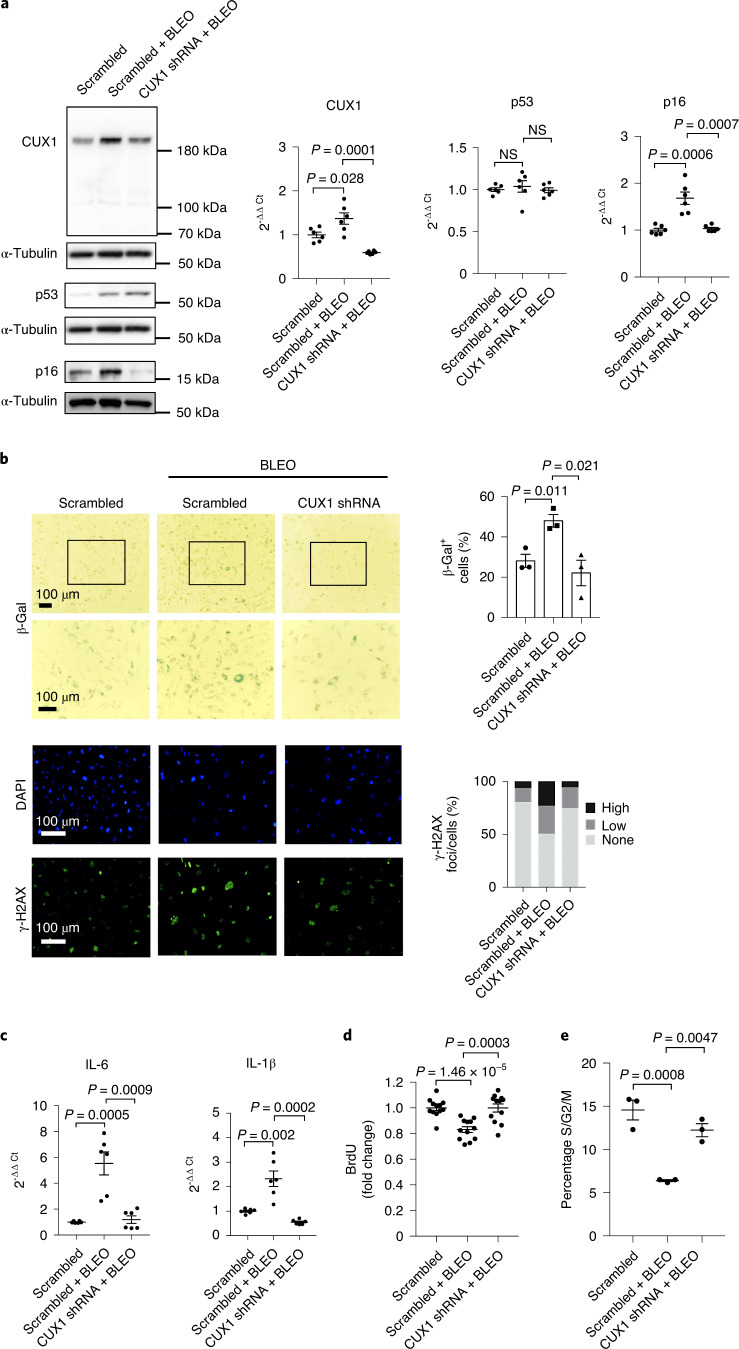
Regulation of bleomycin-induced premature senescence by CUX1. **a**, Immunoblot and qPCR analysis showing induction of CUX1 and p16^INK4a^ in response to bleomycin (BLEO) activation in human ECs (middle). p53 expression was also induced by bleomycin activation, but in a post-transcriptional fashion as evidenced by qPCR. Downregulation of CUX1 by shRNA in human ECs blocked the induction of p16^INK4a^ in response to bleomycin activation, but not p53 (right). Data for immunoblot analysis represent *n* = 3 biologically independent experiments; data for qPCR analysis represent *n* = 3 biologically independent samples, each performed in duplicate. **b**, SA-β-gal (top) and γ-H2AX (bottom) staining demonstrating increase in cellular senescence in bleomycin-treated ECs (middle) and a reduction in senescence in bleomycin-treated and CUX1 shRNA knockdown ECs (right). Right: quantitative plots for both β-gal^+^ cells (%) with SA-β-gal staining and γ-H2AX foci/cells (%) with γ-H2AX staining are shown. Data for SA-β-gal and γ-H2AX staining represent *n* = 3 biologically independent experiments. **c**, qPCR analysis showing increased expression of *IL-6* and in bleomycin-treated ECs (middle) and restoration of their expression following CUX1 shRNA knockdown (right) (*n* = 3). **d**,**e**, Decrease in both BrdU incorporation (**d**) and percentage of S/G2/M cell numbers (**e**) in bleomycin-treated ECs (middle) demonstrated an increase in bleomycin-induced senescence. Knockdown of *CUX1* by shRNA in bleomycin-treated ECs (right) resulted in recovery from decreased BrdU incorporation and reduced percentage of S/G2/M cell numbers, indicating blockage of senescence in bleomycin-treated and CUX1-downregulated human ECs. shCUX1, shRNA for CUX1. Data for BrdU incorporation represent *n* = 12 biologically independent samples; data for cell cycle analysis represent *n* = 3 biologically independent samples. *P* values were calculated using two-tailed Student’s *t*-test, and all data are presented as mean ± s.e.). Source data

**Extended Data Fig. 5:**
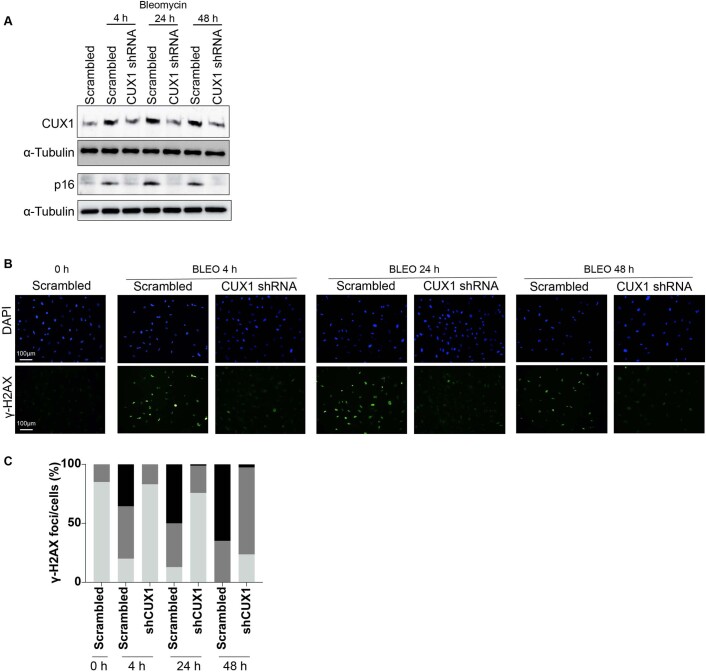
A time course analysis showing that CUX1 knockdown inhibits the *p16*^*INK4a*^-dependent and bleomycin-induced cellular senescence in human ECs. **A**. Western blot analysis showing that knockdown of CUX1 by shRNA inhibits the upregulated p16^INK4a^ expression induced by 0.5 µg/ml bleomycin treatment over a 48 hrs time course. Data for the Western blot analysis represents three biologically independent experiments (n=3). **B**. γ-H2AX staining demonstrating EC senescence induced by bleomycin treatment can be inhibited by downregulation of CUX1 over the same time course. **C**. Quantitative plots showing the γ-H2AX foci/cells (%) in γ-H2AX staining. Data for γ-H2AX staining represents three biologically independent experiments (n=3). Source data

Stress-induced premature senescence is another type of cellular senescence that can be triggered by various stimuli including DNA damage, oxidative stress, oncogene activation and metabolic dysregulation^40–42^. To determine whether CUX1 also plays a role in stress-induced premature senescence, we first investigated protein expression of CUX1 and p16^INK4a^ in ECs treated for 0, 4, 24 and 48 h with bleomycin (0.5 µg ml^–1^), a genotoxic drug known to induce senescence by the introduction of double-stranded DNA breaks^43^. We observed upregulation of both CUX1 and p16^INK4a^ expression at all time points, and a restoration of p16^INK4a^ expression to the level of untreated ECs with CUX1 knockdown by shRNA (Extended Data Fig. 5a). We also observed a corresponding induction of γ-H2AX staining (Extended Data Fig. 5b). Based on these observations, we performed a detailed analysis using human ECs treated with bleomycin (0.5 µg ml^–1^) for 24 h. Both immunoblot and qPCR analysis identified a significant increase in the expression of both CUX1 and p16^INK4a^ in bleomycin-treated ECs (Fig. 5a, left and middle). Bleomycin-treated cells also demonstrated an increase in cellular senescence, as evident by increased staining for both SA-β-gal and γ-H2AX (Fig. 5b, left and middle), increased *IL-6* and *IL-1β* expression (Fig. 5c, left and middle) and reduced BrdU incorporation (Fig. 5d, left and middle) and percentage of cells in S/G2/M phase (Fig. 5e, left and middle). To further demonstrate that CUX1 is required for the induction of this type of premature senescence, we downregulated CUX1 by shRNA in human ECs before bleomycin exposure (Fig. 5a, middle and right). As expected, bleomycin-induced *p16*^*INK4a*^ expression was reduced in CUX1 knockdown ECs (Fig. 5a, middle and right). We also noted inhibition of bleomycin-induced senescence, with a reduction in both SA-β-gal and γ-H2AX staining (Fig. 5b, middle and right), decreased expression of various SASP genes (Fig. 5c, middle and right), increased BrdU incorporation (Fig. 5d, middle and right) and restoration of cell cycle parameters (Fig. 5e, middle and right). Together, these data demonstrate that CUX1 modulates DNA damage-induced premature senescence, presumably via modulation of *p16*^*INK4a*^ expression in ECs.

**Extended Data Fig. 6:**
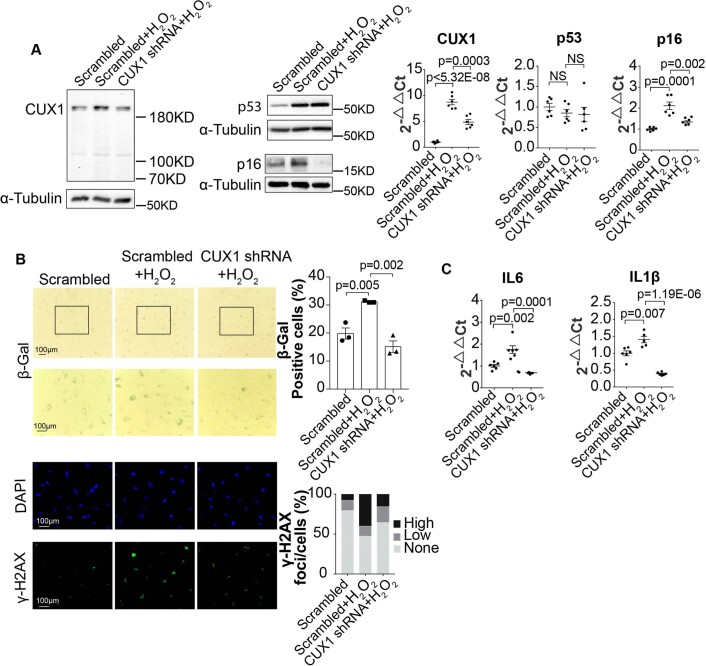
Regulation of H_2_O_2_-induced premature senescence by CUX1. **A**. Western blots and qPCR analysis showing an induction in CUX1 and p16^INK4a^ expression in response to 200 µM H_2_O_2_ exposure for 4 hrs in human ECs (**middle lane**). p53 expression was also induced by H_2_O_2_ activation, but in a post-transcriptional fashion. A downregulation of CUX1 by shRNA in human ECs blocks the induction of p16^INK4a^ in response to the H_2_O_2_ treatment, but not affect the p53 expression (**right lane**). Data for Western blot analysis represents three biologically independent experiments (n=3). Data for qPCR analysis represents three biologically independent experiments (n=3), each performed in duplicate. **B**. SA-β-gal (**upper**) and γ-H2AX staining (**lower**) showing an increase in cellular senescence in H_2_O_2_-treated ECs (**middle panel**) and the restoration of senescence following a CUX1 knockdown (**right panel**) by comparing to the scrambled control (**left panel**). Quantitative plots for both β-gal positive cells (%) in SA-β-gal staining **(upper)** and γ-H2AX foci/cells (%) in γ-H2AX staining (**lower**) are presented on the right side of the panel. Data for SA-β-gal staining and γ-H2AX staining represents three biologically independent experiments (n=3). **C**. qPCR analysis showing an increased expression of the SASP genes *IL-6* and *IL-1β* in the H_2_O_2_-treated ECs (**middle lane**) and the restoration of the expression of these genes upon a CUX1 shRNA knockdown (**right lane**) by comparing to the scrambled control **(left lane**). Data for qPCR analysis represents three biologically independent experiments (n=3), each performed in duplicate. Source data

To extend our findings on the role of CUX1 in regulation of stress-induced premature senescence, we repeated the above assays using exogenous H_2_O_2_ to mimic conditions of oxidative stress^44^. Treatment with H_2_O_2_ (200 µM for 4 h) was noted to induce the expression of CUX1, as well as p16^INK4a^ (Extended Data Fig. 6a, left and middle). These H_2_O_2_-treated ECs exhibited an increase in both SA-β-gal and γ-H2AX staining (Extended Data Fig. 6b, left and middle) as well as induced expression of SASP genes (Extended Data Fig. 6c, left and middle). As expected, shRNA-mediated downregulation of CUX1 expression inhibited H_2_O_2_-induced expression of p16^INK4a^ (Extended Data Fig. 6a, right) and, as a consequence, this further resulted in a reduction of H_2_O_2_-elicited senescent response (Extended Data Fig. 6b,c, right).

### RNA-seq analysis of genes regulated by CUX1

**Extended Data Fig. 7:**
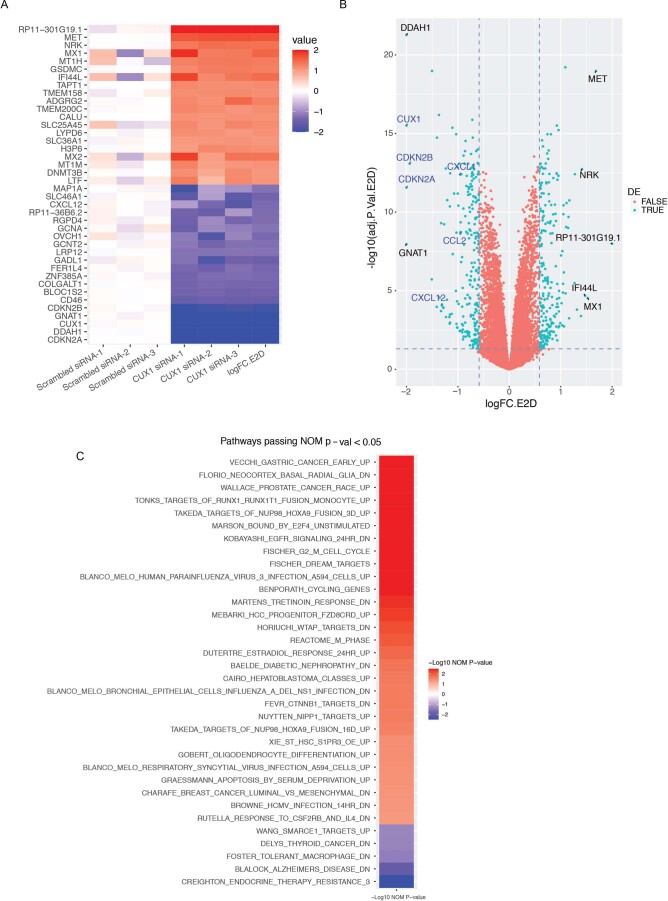
RNA-seq analysis reveals 471 DEGs in CUX1 siRNA-treated human ECs. **A**. Heatmap showing the top 20 upregulated and downregulated DEGs including DDAH1 and DNMT3b 48 hrs after CUX1 was knockdown by siRNA. Gene expression is shown in normalized log2 counts per million. DEGs were selected based on a more than 1.5 FC between CUX1 siRNA-treated samples versus scrambled siRNA-treated samples and adjusted *P*-value less than 0.05. *CUX1* and *p16*^*INK4a*^*, p14*^*ARF*^, and *p15*^*INK4b*^ are among the top 20 downregulated DEGs. **B**. Volcano plot showing 471 DEGs with the log_2_FC in the CUX1 siRNA-treated human ECs. The x-axis displays logFC between CUX1 siRNA-treated samples versus scrambled siRNA-treated samples(logFC.E2D) and the y-axis displays −log10 adjusted *P*-value. Genes with log_2_FC outside the range −2 to +2 were squished to this range. DEG2 in blue including *CDKN2A*, *TNFRSF10B*, *CXCL1*, *CXCL12* and *CCL2* were previously identified as endothelial senescence-related genes^67^. The top 5 upregulated and downregulated DEGs were also listed, they are *CDKN2A*, *CUX1*, *DDAH1*, *GNAT1*, *CDKN2B*, *RP11-301G19.1*, *MET*, *MX1*, *IFI44L*, *NRK*. **C**. List of the 29 upregulated and 5 downregulated pathways with NOM *p*-value < 0.05 in the CUX1 siRNA-treated human ECs.

To further characterize how CUX1 regulates cellular senescence, we performed RNA sequencing (RNA-seq) analysis with total RNA isolated from human ECs at p10 treated with both a scrambled siRNA and a siRNA targeting human CUX1. We estimated that, at p10, approximately 20% of control ECs were senescent and that there was a reduction of ~70% in senescent cells following siRNA targeting of CUX1 (Fig. 3a). As a result, we identified 471 differentially expressed genes (DEGs) with fold change (FC) between the CUX1 siRNA-treated sample and the scrambled siRNA-treated control >1.5 and adjusted *P* < 0.05 (Supplementary Data 3, column D, E and F are scrambled siRNA-treated controls and Column G, H and I are CUX1 siRNA-treated samples). Among these 471 DEGs, we found that 228 genes were upregulated and 243 downregulated in CUX1 siRNA knockdown ECs, with the top 20 upregulated and downregulated DEGs listed in Extended Data Fig. 7a. While we found that *CUX1*, as well as *p14*^*ARF*^, *p16*^*INK4a*^ and *p15*^*INK4b*^, were significantly downregulated (Extended Data Fig. 7b), we detected no obvious alterations in other classical senescence markers, including p53 and HGBM1, nor did we observe classical SASP factors such as IL-6 among these 471 DEGs. This may relate to the relatively modest percentage of overall senescent cells in our cell-based model. Interestingly, we did identify downregulation of dimethylarginine dimethylaminohydrolase-1, an enzyme involved in asymmetric dimethylarginine (ADMA) degradation (Extended Data Fig. 7a). Of note, ADMA is an endothelial nitric oxide synthase (eNOS) inhibitor, and eNOS activity regulates endothelial cell senescence^45,46^. Similarly, we noted upregulation of DNA methyltransferase 3b (Extended Data Fig. 7a), which was previously reported to activate senescence markers including p16^INK4a^ and p21^CIP1/WAF1^ by decreasing the methylation of CpG islands^47^. In addition, gene set enrichment analysis (GSEA) of the 471 DEGs identified 29 upregulated and five downregulated pathways enriched in CUX1 siRNA knockdown human ECs (nominal *P* < 0.025; Extended Data Fig. 7c). These pathways include cell cycle regulation, cancer cell growth, cell differentiation and apoptosis.

### Elevated CUX1 and p16^INK4a^ expression in patients

**Fig. 6:**
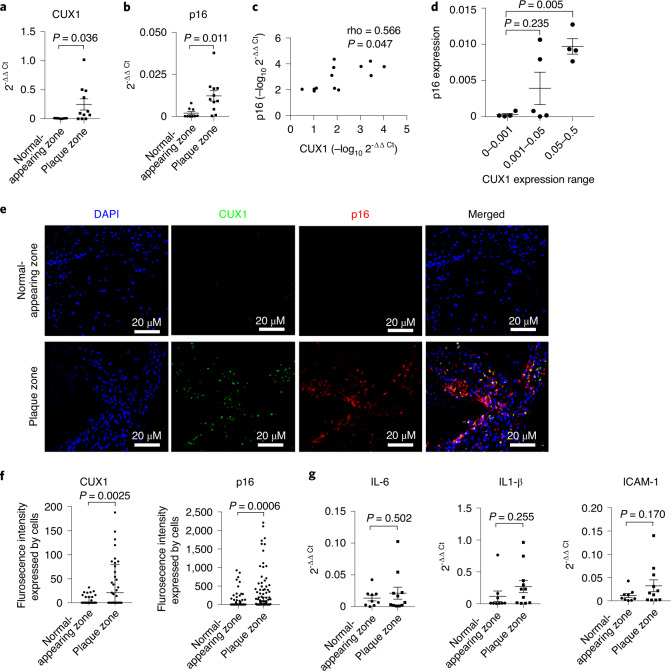
Elevated CUX1 and p16^INK4a^ expression in plaque zones from patients with carotid artery disease. **a**,**b**, qPCR showing significant increase in expression of CUX1 (*P* = 0.036) (**a**) and p16^INK4a^ (*P* = 0.011) (**b**) in plaque versus normal-appearing zones obtained from patients with carotid artery atherosclerosis. Data for qPCR analysis represent *n* = 11 plaque zones and *n* = 9 normal-appearing zones. **c**,**d**, Nonparametric Spearman correlation analysis (**c**) and trend analysis (**d**) showing significant correlation between the expression levels of *CUX1* and *p16*^*INK4a*^ (*P* = 0.047 and *P* = 0.005, respectively; *n* = 13). **e**, Immunocytochemical staining with antibodies specifically against CUX1 (green) and p16^INK4a^ (red) in plaque and normal-appearing zones from patients with carotid artery atherosclerosis. Data were generated by staining of *n* = 8 plaque zone and *n* = 8 normal-appearing zones in two independent experiments. DAPI (blue) was applied to stain fixed cells. **f**, Statistical analysis of immunocytochemical staining showing significant induction of CUX1 (*P* = 0.0025) and p16^INK4a^ (*P* = 0.0006) in plaque zones compared to normal-appearing zones. **g**, qPCR showing a trend of increase with no statistical significance in the expression of SASP genes *IL-6* (left; *P* = 0.502), *IL-1β* (middle; *P* = 0.255) and *ICAM1* (right; *P* = 0.17). Data for qPCR analysis represent *n* = 11 plaque zones and *n* = 9 normal-appearing zones. **a**–**d**,**g**, Data presented as mean ± s.e. *P* values were calculated using two-tailed Student’s *t*-test. **f**, Non-normally distributed data are presented as median ± interquartile range, and *P* values were calculated with the nonparametric Mann–Whitney test. Source data

Previous studies have established that depletion of *p16*^*INK4a*^-positive senescent cells by genetic manipulation or pharmacological strategies can delay the onset of age-related diseases, as well as extend longevity^9,14,15,48^. Based on this association we hypothesized that, as a direct regulator of *p16*^*INK4a*^, CUX1 expression might be upregulated in patients with age-related diseases such as atherosclerosis. To test this, human atherosclerotic plaques were obtained from patients undergoing carotid endarterectomy. Total RNA was isolated from both plaques and zones of normal appearance, and expression of *CUX1* measured by qPCR. A significant induction in *CUX1* expression was observed in plaque zones compared to normal zones (*P* = 0.036) (Fig. 6a). Consistent with this induction of *CUX1*, as well as a previous publication showing *p16*^*INK4a*^ is abundantly expressed in atherosclerotic lesions^49^, we also detected a significant increase in *p16*^*INK4a*^ expression with *P* = 0.011 in the plaque zones (Fig. 6b). Even with these limited numbers of samples, both Spearman correlation and trend analysis identified a significant correlation between the expression levels of *CUX1* and *p16*^*INK4a*^ with P = 0.012 and 0.005, respectively (Fig. 6c,d). However, due to the limited number of samples, no significant association could be identified between the expression level of *p16*^*INK4a*^ and fSNP rs1537371 genotypes. To confirm these results, we also performed immunocytochemical staining using antibodies specifically against CUX1 and p16^INK4a^. Only CUX1 or p16^INK4a^ staining colocalized with DAPI staining was used to calculate for fluorescence intensity. A similar induction of both CUX1 (green) and p16^INK4a^ (red) was observed by comparison of plaque zones to normal zones (Fig. 6e), and was quantitatively evaluated using the nonparametric Mann–Whitney test for pairwise comparisons with *P* = 0.0025 for CUX1 (*n* = 8) and *P* = 0.0006 for p16^INK4a^ (*n* = 8) (Fig. 6f). In addition, we also checked the expression of SASP genes *IL-6*, *IL-1β* and *ICAM1*. All three inflammatory markers showed a trend towards elevation in plaque zones, although with limited sample size none reached statistical significance (Fig. 6g). These data, together with our other findings, suggest that stress-induced upregulation of CUX1 may promote atherosclerosis by induction of cellular senescence through modulation of *p16*^*INK4a*^ expression.

### CUX1 regulates cellular senescence independently of p53

**Fig. 7:**
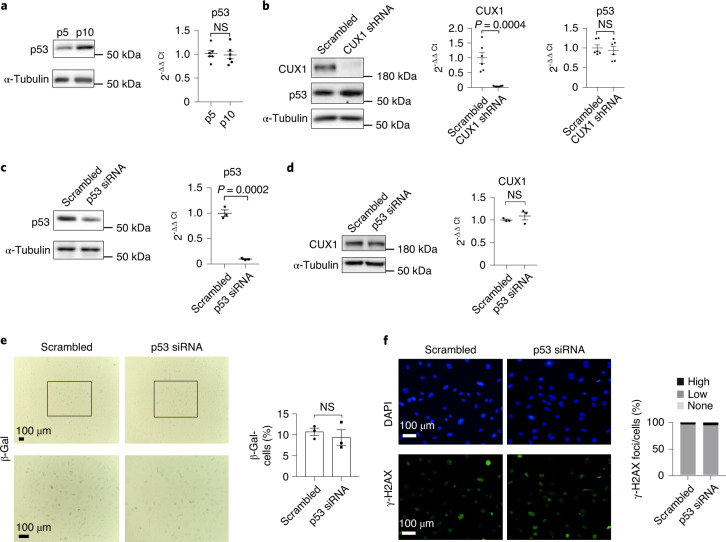
Demonstration that CUX1 regulates replicative senescence independently of p53 expression. **a**, Immunoblot analysis showing passage-dependent induction of p53 expression in p10 compared to p5 ECs. qPCR analysis showing that induction of *p53* was not at the transcriptional level. **b**, Immunoblot (left) and qPCR analysis (right) showing no significant change in p53 expression following shRNA-mediated CUX1 knockdown in p10 ECs. **c**,**d**, Immunoblot and qPCR analysis showing no significant change in CUX1 expression (**d**) after p53 siRNA-mediated knockdown (**c**) in human ECs. **e**,**f**, SA-β-gal (**e**) and γ-H2AX (**f**) staining showing no significant change in EC senescence by comparison of scrambled siRNA control ECs with p53 siRNA knockdown ECs. Data for immunoblot analysis represent *n* = 3 biologically independent experiments; data for qPCR analysis represent *n* = 3 biologically independent samples, each performed in duplicate. Data for SA-β-gal and γ-H2AX staining represent *n* = 3 biologically independent experiments. *P* values were calculated using two-tailed Student’s *t*-test, and all data are presented as mean ± s.e.). Source data

As we know, both replicative and stress-induced premature senescence are mediated through the p53/p21 and/or p16^INK4a^ /RB pathways^17,20,22,50^. To determine whether p53 also plays a role in the induction of cellular senescence in ECs, we first checked its expression in p5 and p10 ECs, as well as in ECs treated with both bleomycin and H_2_O_2_. We detected an elevated level of p53 expression in p10 ECs versus p5 ECs (Fig. 7a), as well as in bleomycin- and H_2_O_2_-treated ECs (Fig. 5a, left and middle and Extended Data Fig. 6a, left and middle). While these data are consistent with previous reports^51,52^, unexpectedly increased expression of p53 could be detected only at the protein level by immunoblot, but not at the mRNA level by qPCR analysis (Figs. 7a and 5a and Extended Data Fig. 6a). This suggests that regulation of p53 expression under these conditions is post-transcriptional. To check whether CUX1 regulates p53 expression in human ECs, we performed shRNA knockdown of CUX1 in p10 ECs. No significant change in p53 expression was observed in these ECs (Fig. 7b). We also did not detect any change in p53 expression when CUX1 was reduced by shRNA knockdown in ECs treated with either bleomycin (Fig. 5a, middle and right) or H_2_O_2_ (Extended Data Fig. 6a, middle and right), suggesting that CUX1 regulates cellular senescence independently of p53, at least in ECs.

In addition, we also checked the possibility that p53 regulates CUX1 expression in human ECs which, in turn, regulates cellular senescence. As a result, no alteration in CUX1 expression was detected in p53 siRNA knockdown ECs (Fig. 7c,d). Also, no change in cellular senescence was observed between scrambled control ECs and p53 siRNA knockdown ECs, in terms of either SA-β-gal (Fig. 7e) or γ-H2AX staining (Fig. 7f).

## Discussion

**Fig. 8:**
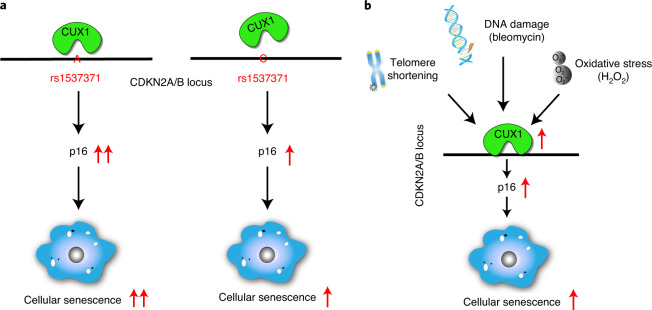
Models highlighting the role of CUX1 in mediation of cellular senescence by activation of *p16*^*INK4a*^ expression. **a**, The mechanism underlying the contribution of atherosclerosis-associated fSNP rs1537371 to susceptibility to age-related disease. Increased binding of CUX1 to the A allele (risk allele) versus the C allele (nonrisk allele) resulted in a higher level of *p16*^*INK4a*^ expression which, in turn, augments senescence. **b**, The mechanism underlying the contribution of the CUX1/p16^INK4a^ pathway to cellular senescence. Increased expression of CUX1 in response to telomere shortening, DNA damage and oxidative stress resulted in upregulated expression of *p16*^*INK4a*^ and induction of cellular senescence.

In this report, we demonstrate that CUX1 regulates both replicative and stress-induced senescence in ECs by activation of the expression of p16^INK4a^, a known regulator of senescence induction^9,14,15^. This activation depends on the allele-imbalanced binding of CUX1 to fSNP rs1537371, with the risk allele A binding more CUX1 than the nonrisk allele C. Increased binding of CUX1 to rs1537371 induced a higher level of *p16*^*INK4a*^ expression, which resulted in an increase in cellular senescence. Since accumulation of senescent cells has been detected in atherosclerotic plaques in patients with atherosclerosis^53,54^, we believe that our findings provide a potential pathophysiological mechanism that explains the contribution of fSNP rs1537371 to atherosclerotic risk (Fig. 8a).

Consistent with the genetic data described above, we detected increased expression of CUX1 in ECs in a passage-dependent fashion, and following treatment with DNA-damaging agents or oxidative stress. We further demonstrate that induction of CUX1 by all the factors above can induce cellular senescence by activation of *p16*^*INK4a*^, regardless of the genotype of fSNP rs1537371. We believe that these findings reveal a mechanism implicating CUX1 as an inducer of cellular senescence and, hence, as a potential driver of age-related diseases such as atherosclerosis (Fig. 8b).

In addition, our data suggest that CUX1-induced DNA damage, as detected by γ-H2AX, is *p16*^*INK4a*^ dependent. In particular, we observed that knockdown of *p16*^*INK4a*^ in CUX1-overexpressed ECs rescued the DNA damage phenotype (Figs. 4 and 5) while overexpression of *p16*^*INK4a*^ in CUX1 knockdown restored γ-H2AX staining (Extended Data Fig. 3). These data support the hypothesis that the effect of CUX1 on the DNA damage response is probably mediated through *p16*^*INK4a*^. Unfortunately, precisely how *p16*^*INK4a*^ affects DNA repair is not well understood^55^. Of note, it was recently reported that upregulation of *p16*^*INK4a*^ decreases both nucleotide and deoxyribonucleotide synthesis and induces oncogene-induced senescence, probably by blocking the repair of DNA damage^56^. However, we cannot exclude the possibility that CUX1 directly affects DNA repair, as previously reported^57,58^.

Collectively, in this report, we demonstrate how to potentially utilize post-GWAS functional studies to obtain important biological insights. In particular, our data reveal a new mechanism underlying the contribution of CUX1/rs1537371 in the pathogenesis of, and/or susceptibility to, aging and age-related diseases. However, to fully understand the role played by CUX1 in the regulation of cellular senescence and senescence-induced, age-related diseases, a comprehensive in vivo functional study with a *CUX1* null mouse model would be warranted.

## Methods

### Cell culture and reagents

Primary human arterial ECs (catalog no. CC-2535) and human arterial VSMCs (catalog no. CC-2571) were purchased from Lonza. ATCC supplied 293T (catalog no. ATCC CRL-3216), THP1 (catalog no. ATCC TIB-202) and HMC3 (catalog no. ATCC CRL-3304) cells. All cells were free of mycoplasma without authentication. ECs were cultured in basal medium EGM-2, VSMCs in SmGM-2 supplemented with Bullet Kit (Lonza) and HMC3 in DMEM/F12 mix (1:1) supplemented with 10% fetal bovine serum (FBS). All cells were cultured at 37 °C in 5% CO_2_.

### Primers and antibodies

All primers used in this study were purchased from IDT and are listed in Supplementary Table 2. All antibodies used are listed in Supplementary Table 3, with corresponding supplier information.

### Isolation of atherosclerotic plaques

Atherosclerotic plaques were obtained from patients undergoing carotid endarterectomy at the Department of Surgery at UPMC Presbyterian Hpospital. The use of human materials was approved by the University of Pittsburgh (Institutional Review Board no. STUDY18100138), and written informed consent was obtained from all individuals before operative procedures.

### Reel-seq

To identify fSNPs at the *CDKN2A/B* locus, a Reel-seq library containing both alleles of the 193 SNP sequences was built with the construct sequence shown in Supplementary Table 2. The library was amplified and regenerated by primers, sequecing and G3 with Accuprime Taq polymerase (Invitrogen). For screening, ~10 µg of NE isolated from PBMC cells (buffer for control) was mixed with ~50 ng of library DNA using the binding buffer provided with the LightShift Chemiluminescent EMSA Kit (Thermo Fisher Scientific), and subsequently incubated at room temperature (RT) for 2 h. The reaction was performed in triplicate, with three buffer-treated controls and three NE-treated samples. All samples were resolved on a 6% TBE native gel for gel shifting. After completion of electrophoresis, unshifted bands from each of the controls and samples were cut and isolated. The isolated library DNA was next amplified by PCR using sequecning and G3 primers, and regenerated libraries were used for the next round of gel shifting. In total, seven rounds were performed. After screening, standard Illumina amplicon sequencing was performed with the PCR product from rounds 1, 4 and 7 (refs. ^28,59^).

### FREP–MS

FREP–MS assay was performed as previously described^28^. In brief, ~10 µg of FREP construct DNA (Supplementary Table 2), either for samples or controls, was conjugated to 150 µl of streptavidin-coupled Dynabeads (Life Technologies) according to the manufacturer’s instructions. DNA beads were then washed and mixed with 1 mg of NE isolated from human ECs cells at RT for 1 h. After separation and washing, protein-DNA beads were digested with 5 µl of EcoR I (100 units µl^–1^, NEB) at 37 °C for 30 min to remove the 3’DNA plus proteins bound to this DNA fragment. After separation and washing, protein-DNA beads were subsequently digested with 5 µl of BamH I (100 units µl^–1^, NEB) at 37 °C for 45 min to release the fSNP sequence plus fSNP-bound proteins. The supernatant was then run on an 8% short SDS–polyacrylamide gel electrophoresis (SDS–PAGE) gel (http://www.bidmcmassspec.org/), then collected for protein complex identification by mass spectrometry. To identify fSNP-bound proteins, all proteins with peptide counts in both samples and controls were eliminated. fSNP-bound proteins were identified as those with peptide counts that appeared only in the sample but not in the control.

### EMSA

EMSA was performed using the LightShift Chemiluminescent EMSA Kit (Thermo Fisher Scientific) according to the manufacturer’s instructions. For probe, a 31-base pair (bp), SNP-centered fragment was made by annealing two oligos. Double-stranded oligos were then biotinylated using the Biotin 3’ End DNA Labeling Kit (Thermo Fisher Scientific). NE was isolated from human ECs. After incubation of DNA and NE at RT for 30 min, the DNA–NE complex was resolved on 6% TBE native gel for mobility shifting. Data represent *n* = 3 independent biological replicates.

### Luciferase reporter assay

Luciferase reporter assays were performed in 293T cells using the pGL3-Promoter vector (Promega, catalog no. E1761). Next, 31-bp, SNP-centered fragments were cloned into Sac I and Xho I sites in the pGL3-Promoter vector. For control, an irrelevant 31-bp DNA fragment was cloned into the same vector. The same amounts of both SNP and control construct were transfected into 293T cells by FuGENE HD transfection reagent (Promega), together with the same amount of control vector, pRL-TK, which provides constitutive expression of *Relnlla* luciferase (Promega). Luciferase reporter activity was measured by normalization of firefly luciferase reporter activity to *Renilla* luciferase activity using the Dual-Glo Luciferase Reporter Assay System (Promega). All experiments were performed according to the manufacturer’s protocol. Data represent *n* = 6 independent biological replicates.

### CRISPR–Cas9 genome editing

CRISPR–Cas9 was performed using the LentiCRISPR v.2 vector system (Addgene). Lentiviruses were infected into the human microglia cell line HMC3. Single, puromycin-resistant clones were selected using limited-dilution cloning in 96-well plates. Genomic DNA was isolated from each clone, and DNA fragments crossing fSNP rs1537371 were amplified and sequenced. Cells positive for mutations, except for homozygous mutations, were subcloned and the same DNA fragments were cloned into pGEM-T Easy vector (Promega) for sequencing of both alleles. For control, we used polyclonal cells targeted by the same CRISPR–cas9 targeting vector containing a guide RNA sequence irrelevant to rs1537371.

### ChIP assay

ChIP was performed as described previously^60^. Briefly, scrambled control shRNA-infected human ECs and CUX1 shRNA knockdown ECs were crosslinked with 1% formaldehyde for 10 min. Sonication was carried out at 30% amplitude, with 20 s on and 50 s off for 5 min, followed by overnight incubation of 10 μg of anti-CUX1 antibody coupled to Dynabeads Protein A/G (Thermo Fisher Scientific, catalog nos. 10001D and 10003D) with sonicated samples at 4 °C. DNA pulled down by antibody, and input DNA, were purified with the Qiagen PCR purification kit after reversal of the crosslink. Purified DNAs were used for qPCR analysis of the DNA fragment containing fSNP rs1537371 with primers ChIP-F and ChIP-R (Supplementary Table 2). ChIP results were measured by normalization of ChIP DNA to input DNA. For antibody isotype control, a rabbit anti-IgG antibody was used; two randomly selected DNA regions were used as negative controls. For sequencing, PCR products from input and ChIP DNA in the scrambled shRNA control were cloned into pGEM-T Easy vector (Promega) and sequenced by Sanger sequencing. Data represent a combination of *n* = 3 independent samples.

### AIDP–Wb analysis

AIDP–Wb was performed as previously described^29^. In brief, a 31-bp biotinylated SNP sequence centered with either the risk or nonrisk allele was generated by annealing two biotinylated primers (IDT). Approximately 1 µg of DNA was then attached to 40 µl of Dynabeads M-280 Streptavidin. DNA beads were mixed with ~100 µg of NE isolated from ECs at RT for 1 h, with rotation. After washing off unbound proteins, DNA-bound proteins were eluted using sample buffer and resolved on an SDS–PAGE gel for immunoblot analysis using an antibody directed against CUX1. For an internal loading control, the same blot was probed using an antibody directed against PARP-1. Data represent *n* = 3 independent biological replicates.

### qPCR analysis

Total RNA was isolated with the RNeasy Mini kit (Qiagen). Complementary DNA was synthesized with SuperScript III Reverse Transcriptase (Invitrogen) after treatment of RNA samples with DNase I (Invitrogen). All procedures were performed following the manufacturer’s protocols. qPCR was operformed with the StepOne real-time PCR system according to the protocol for the Power SYBR Green PCR Master Mix (Applied Biosystems) and for TaqMan Universal PCR Master Mix (Applied Biosystems). The following probe/primer mixes for TaqMan PCR were purchased from Applied Biosystems: CUX1 Hs00738851_m1; *p14*^*ARF*^ Hs99999189_m1; *p15*^*INK4b*^ Hs00793225_m1; *p16*^*INK4a*^ Hs02902543_mH; ANRIL Hs04259472_m1; and GAPDH internal control (Hs02786624_g1). Other primers used are listed in Supplementary Table 2. Data represent the combination of *n* = 3 independent samples.

### Immunoblot analysis

Whole-cell lysates were prepared using RIPA buffer (Sigma). Cytosolic proteins and nuclear proteins were isolated with NE-PER Nuclear and Cytoplasmic Extraction Reagents (Thermo Scientific) according to the manufacturer’s instructions. Proteins were resolved on SDS–PAGE gels and transferred to polyvinylidene difluoride membranes. Proteins were detected with gene-specific antibodies. All antibodies were purchased and used as listed in Supplementary Table 3. For a loading control, α-tubulin was used. Data represent *n* = 3 independent biological replicates.

### Senescence β-gal staining

The Senescence β-Galactosidase Staining Kit (Cell Signaling) was used to stain senescent cells. Visualization was performed using an RVL-100-G microscope (Echo Laboratories). Images were analyzed with ImageJ software (v.1.52K, NIH). Data represent *n* = 3 independent biological replicates.

### γ-H2AX staining

Cells were plated on glass coverslips and fixed in 4% paraformaldehyde. For γ-H2AX staining, cell membranes were solubilized in PBS containing 5% FBS and 0.5% Triton X-100. Cells were first incubated with γ-H2AX antibodies in solubilizing buffer for 1 h, and immunofluorescence was detected with Alexa Fluor 488-conjugated secondary antibody. Cells were counterstained with DAPI (Sigma, catalog no. D9542). Visualization was done using an RVL-100-G microscope (Echo Laboratories). Images were analyzed using ImageJ software (v.1.52K, NIH). Data represent *n* = 3 independent biological replicates.

### Telomere length quantification

Genomic DNA was extracted from human ECs. The Absolute Human Telomere Length Quantification qPCR Assay Kit (ScienCell, catalog no. 8918) was used to measure telomere length. Data represent a combination of *n* = 3 independent samples.

### BrdU proliferation assay

Human EC proliferation was determined by BrdU incorporation using the BrdU Cell Proliferation Assay Kit (Cell Signaling, catalog no. 6813). Briefly, human ECs were incubated with lentivirus(es) for 48 h. Cells were then subcultured (10,000 cells per well) in 96-well plates for 24 h with or without 0.5 μg ml^–1^ bleomycin, then 1× BrdU was added to the culture medium for DNA labeling. The labeling medium was removed after 2 h, then cells were fixed and DNA was denatured by the addition of 100 μl of fixing/denaturing solution for 30 min. The incorporated BrdU was then detected by a mouse anti-BrdU monoclonal antibody and measured using an anti-mouse IgG, horseradish peroxidase-linked antibody following the manufacturers’ instructions. Data for the BrdU proliferation assay represent *n* = 12 independent biological samples.

### Flow cytometry

Flow cytometry analysis was performed as previously reported^61^. In brief, human ECs cultured in 12-well plates were infected with lentivirus(es) for 48 h. Cells were then incubated for 24 h in fresh medium with or without 0.5 μg ml^–1^ bleomycin and collected by trypsin. After washing in FACS buffer (PBS containing 5% bovine serum albumin (BSA)), cells were permeabilized with the FoxP3 permeabilization kit (eBioscience). Cell cycle analysis was performed with anti-Ki67 antibody and propidium iodide (eBioscience) staining. Data were acquired and analyzed using a Fortessa Flow Cytometer (Becton Dickinson) and FlowJo software (Tree Star). Data for flow cytometry analysis represent *n* = 3 independent biological samples.

### siRNA knockdown

For siRNA transient knockdown in human ECs, siRNAs for human *CUX1*, *p53*, *p15*^*INK4b*^ and ANRIL were purchased from Horizon Discovery and knockdown was performed according to the manufacturer’s protocol. siRNAs for *p14*^*ARF*^ were purchased from IDT, and the sequence of sense and antisense RNAs is listed in Supplementary Table 2. For *CUX1* and *p16*^*INK4a*^ shRNA knockdown in human ECs, VSMCs and 293T cells, lentiviruses were generated using pLKO.1 puro vector (Addgene). Forty-eight hours after infection, cells from shRNA knockdown and scrambled controls were collected for different assays. The shRNA targeted sequences are listed in Supplementary Table 2.

### Overexpression of CUX1 and p16^INK4a^

For overexpression of human CUX1 p200, human *CUX1* cDNA from pXJ42-p200 CUX1 (Addgene) was cloned into the lentiviral expression vector pLVX puro using Xho I and Xba I cutting sites (Takara Bio) and confirmed by sequencing. p16^INK4a^ was overexpressed using lentiviral expression vector p156RRL (Addgene). Lentiviruses were generated by transfection of 293T cells and used to infect human ECs.

### RNA-seq

Total RNA was extracted from scrambled siRNA knockdown and *CUX1* siRNA knockdown ECs at p10 using the RNeasy Mini kit (Qiagen, catalog no. 74104). All samples were quantified and assayed to confirm minimum RNA integrity number of at least 9.3 using an Agilent Bioanalyzer (High Sensitivity DNA Kit, catalog no. 5067-4626). Next, 1 µg of total RNA per sample underwent mRNA capture and was then fragmented at 94 °C for 6 min. Sequencing libraries were prepared according to the manufacturer’s protocol using ten cycles of final amplification (KAPA mRNA HyperPrep Kit, catalog no. KK8580 and KAPA UDI Adapter Kit, catalog no. KK8727). Next-generation sequencing was performed on an Illumina NextSeq500 (75-bp paired end) to a targeted depth of ~20 million reads per sample.

### RNA-seq data analysis

Paired-end sequencing reads (75 bp) were mapped using STAR to human genome assembly 38 (Hg38) with gencode v.38 annotation^62^. Genes with at least three sample counts >20 raw reads were then analyzed using the R Limma Voom library^63^ RNA-seq quantitation pipeline. We defined linear contrast as: difference between CUX1 siRNA-treated samples versus scrambled siRNA-treated samples, normalized raw counts using the default Voom mean-variance transformation and finally calculated log_2_FC and adjusted *P* values. DEGs were defined as genes with Benjamini–Hochberg-corrected *P* < 0.05 and fold change >1.5 in the linear contrast between CUX1 siRNA-treated samples versus scrambled siRNA-treated samples.

Differentially expressed genes were analyzed using Gene Set Enrichment Analysis 4.1 (GSEA)^64^. GSEA preranked analysis was performed with default parameters, including 1,000 permutations, minimum size 15, maximum size 500, normalization mean div and human gene symbol platform. GSEA input genes were ranked by log_2_FC, and their enrichment scores were calculated using the MSigDB7.0 C2 collection of expert curated gene sets. We defined significant gene sets as those with GSEA nominal *P* < 0.05 in either enriched or depleted C2 pathways.

### Immunocytochemical staining for p16^INK4a^ and CUX1

Human atherosclerotic plaques were obtained from patients undergoing carotid endarterectomy. The part of the carotid artery showing hard, calcified tissue was used as a plaque zone while the part far from the calcified zone was used as the normal-appearing zone. Both plaque and normal-appearing zones were separated and fixed in 4% buffered formalin for 2 h and stored in 30% sucrose solution containing 0.05% sodium azide overnight. Sections of 10-µm thickness were permeabilized with 0.1% triton X-100 for 4 h and blocked overnight in PBS containing 2% BSA in 96-well cell culture plates. Sections were incubated for a further 24 h with primary antibodies against p16INK4a *p16* (Invitrogen, catalog no. MA5-17142, 1:500 dilution) and CUX1 (Proteintech, catalog no. 11733-1-AP, 1:100 dilution). After washing with PBS, sections were incubated for 1 h at RT with fluorochrome-conjugated secondary antibodies (Alexa Fluor 488 goat anti-mouse and Alexa Fluor 647 goat anti-rabbit). Tissue sections were stained and mounted with VECTASHIELD DAPI, and images were taken using confocal laser microscopy and analyzed with imageJ. The data represent two independent experiments, with *n* = 8 plaque zones and *n* = 8 eight normal-appearing zones.

### Datasets for comparison and visualization of fSNPs

The University of California, Santa Cruz genome browser was used to visualize data and create genomic view snapshots for regulatory regions of CDKN2A/B^65^.

#### Enhancer and promoter prediction

Enhancer and promoter prediction were performed using the GeneHancer database^66^.

#### DNase I hotspot

We used the track of DNase I Hypersensitivity on Human Astrocytes-spinal cord from ENCODE.

#### Histone marker

The layered H3K4Me1 and layered H3K27Ac tracks show where modification of histone proteins is suggestive of enhancer and, to a lesser extent, other regulatory activity. The layered H3K4Me3 track shows a histone mark associated with promoters^31^. We used the tracks of markers H3K27ac, H3K4me1 and H3K4me3 on HUVEC.

#### Transcription factor ChIP–seq data

This track shows DNA regions where transcription factors, and proteins responsible for modulation of gene transcription, bind—as assayed by ChIP—with antibodies specific to the transcription factor, followed by sequencing of the precipitated DNA (ChIP–seq). We used the tracks of CUX1 on GM12878, K562 and MCF-7.

### Statistics and reproducibility

For normally distributed data, all data are presented as mean ± s.e. *P* values were calculated using two-tailed Student’s *t*-test. Non-normally distributed data relating to quantification of *p16*^*INK4a*^ expression in Fig. 1h and CUX1 and p16^INK4a^ immunocytochemical staining in Fig. 6f are presented as median ± interquartile range, and *P* values were calculated with the nonparametric Mann–Whitney test for pairwise comparison. All data presented in this work are reproducible. No statistical method was used to predetermine sample size. No data were excluded from the analyses. The experiments were not randomized, and no blinding was applied to allocation during experiments and outcome assessment except for phenotype–genotype analysis in Fig. 1h.

### Reporting Summary

Further information on research design is available in the Nature Research Reporting Summary linked to this article.

## Supplementary information

Supplementary Tables 1–3.

Reporting Summary

Supplementary Data 1 Reel-seq DNA sequencing data.

Supplementary Data 2 Peptide spectrum count data.

Supplementary Data 3 List of 471 DEGs from RNA-seq analysis.

## Data Availability

Reel-seq data, FREP–MS data and differentially expressed genes identified by RNA-seq are provided in Supplementary Data 1, 2 and 3. RNA-seq sequencing data have been deposited in GEO with accession code GSE186528. All other data are available from the corresponding author upon reasonable request.

## Source data

Source Data Fig. 1 Unprocessed numerical data.
Source Data Fig. 2 Unprocessed numerical data.
Source Data Fig. 3 Unprocessed numerical data.
Source Data Fig. 4 Unprocessed numerical data.
Source Data Fig. 5 Unprocessed numerical data.
Source Data Fig. 6 Unprocessed numerical data.
Source Data Fig. 7 Unprocessed numerical data.
Source Data Extended Data Fig. 1 Unprocessed extended data numerical data.
Source Data Extended Data Fig. 2 Unprocessed extended data numerical data.
Source Data Extended Data Fig. 3 Unprocessed extended data numerical data.
Source Data Extended Data Fig. 4 Unprocessed extended data numerical data.
Source Data Extended Data Fig. 5 Unprocessed extended data numerical data.
Source Data Extended Data Fig. 6 Unprocessed extended data numerical data.
Source Data gels and blots. Data Fig. 1 Unprocessed gels and blots.
Source Data gels and blots. Data Fig. 2 Unprocessed gels and blots.
Source Data gels and blots. Data Fig. 3 Unprocessed gels and blots.
Source Data gels and blots. Data Fig. 4 Unprocessed gels and blots.
Source Data gels and blots. Data Fig. 5 Unprocessed gels and blots.
Source Data gels and blots. Data Fig. 7 Unprocessed gels and blots.
Source Data Extended Data gels and blots. Fig. 3 Unprocessed extended data gels and blots.
Source Data Extended Data gels and blots. Fig. 4 Unprocessed extended data gels and blots.
Source Data Extended Data gels and blots. Fig. 5 Unprocessed extended data gels and blots.
Source Data Extended Data gels and blots. Fig. 6 Unprocessed extended data gels and blots.
