# Supplementary material for: Post-GWAS functional analysis identifies CUX1 as a regulator of p16INK4a and cellular senescence
Source: Nat Aging. 2022 Feb 17;2(2):140–54. doi: 10.1038/s43587-022-00177-0 (PMC10154215; doi:10.1038/s43587-022-00177-0)
Supplement: Supplementary file 1 — Supplementary Tables 1–3. [file 43587_2022_177_MOESM1_ESM.pdf]

nature portfolio

https://doi.org/10.1038/s43587-022-00177-0

Supplementary information

Post-GWAS functional analysis identifies CUX1 as a regulator of p16^{INK4a} and cellular senescence

In the format provided by the authors and unedited

Supplementary Table 1. Protein peptide spectrum counts showing proteins identified by FREP-MS binding to the fSNP rs1537371 on the *CDKN2A/B* locus.

Protein name	Accession number	Spectrum count	
		Control*	rs1537371
CUX1	P39880	0	9
SATB1	Q01826	0	8
SATB2	Q9UPW6	0	5
HOXA10	P31260	0	4
NFIC	P08561-2	0	2
MYH9	P35579	0	2

Supplementary Table 2. Primers used in this paper

Usage	Primer name	Sequence
QPCR	IL6-F	GCAGAAAACAACCTGAACCTT
	IL6-R	ACCTCAAACTCCAAAAGACCA
	IL1b-F	ACAGATGAAGTGCTCCTTCCA
	IL1b-R	GTCGGAGATTCGTAGCTGGAT
	ICAM1-F	AGCGGCTGACGTGTGCAGTAAT
	ICAM1-R	TCTGAGACCTCTGGCTTCGTCA
	GAPDH-F	CGACCACTTTGTCAAGCTCA
	GAPDH-R	AGGGGTCTACATGGCAACTG
shRNA knockdown	CUX1 shRNA (338-358)	GCACGATATTGAAACAGAGAA
SDCP	SDCPbio371A-F	GTCTGTGTTCCGTTGTCCGTGCTGAATGGATCCGGATCCGGCATGTTATAATTTAATTGGCAGCATTATTGAATTCGAATTCGC
	SDCP371A-R	GCGAATTCGAATTCAATAATGCTGCCAATTAAATTATAACATGCCGGATCCGGATCCATT
	SDCP371A-F-7D	GTCTGTGTTCCGTTGTCCGTGCTGAATGGATCCGGATCCGGCATGTTATAAGGCAGCATTATTGAATTCGAATTCGC
	SDCP371A-R-7D	GCGAATTCGAATTCAATAATGCTGCCTTATAACATGCCGGATCCGGATCCATT
Luciferase reporter	Luciferase reporter insert rs1537371-A	GGCATGTTATAATTTAATTGGCAGCATTATT
	Luciferase reporter insert rs1537371-C	GGCATGTTATAATTTCATTGGCAGCATTATT
	Luciferase reporter insert rs1537371-T	GGCATGTTATAATTTTATTGGCAGCATTATT
CRISPR/CAS9	gRNA-rs1537371-F	CACCGATGGCATGTTATAATTTAAT

	gRNA- rs1537371-R	AAACATTAAATTATAACATGCCATC
Reel-seq	Seq	TGCTCGGGGATCCAGGAATTC/CTGGAG/31 bp SNP sequence/CTCCAG/GGATGACGACGATAAGCTCG
	G3	CGAGCTTATCGTCGTCATCC
	Reel-seq library construct	TGCTCGGGGATCCAGGAATTC/CTGGAG /31 bp SNP sequence/ CTCCAG/GGATGACGACGATAAGCTCG
ChIP	ChIP-F	GGGGAGCTGGGTTTGATAGC
	ChIP-R	TGGGGGCCCTTAAGAGACAA
siRNA	*p14^{ARF}*	AUCAGCACGAGGGCCACAGCGGCGG

Supplementary Table 3. Antibodies used in this paper

Antibody	Vendor	Cat#	Usage	Dilution/ amount	Validation	Monoclonals
CUX1	Sigma	ABE217	WB, ChIP	1:1000, 10ul	https://www.sigmaaldrich.com/US/en/product/mm/abe217?context=product	Polyclonal
CUX1	Proteintech	11733-1AP	ICC	1:100	https://www.ptglab.com/products/CUX1-Antibody-11733-1-AP.htm	Polyclonal
*p14^ARF^*	Invitrogen	MA5-14260	WB	1:150	https://www.thermofisher.cn/cn/zh/antibody/product/p14ARF-Antibody-clone-14P02-DCS-240-Monoclonal/MA5-14260	Monoclonal
*p15^INK4b^*	Invitrogen	PA5-49749	WB	1:1000	https://www.thermofisher.cn/cn/zh/antibody/product/CDKN2B-Antibody-Polyclonal/PA5-49749	Polyclonal
*p16^INK4a^*	Proteintech	10883-1-AP	WB	1:1000	https://www.ptglab.co	Polyclonal

					m/products/P16,P19-Antibody-10883-1-AP.htm	
p16^{INK4a}	Invitrogen	PA1-9025	ICC	1:100	https://www.thermofisher.cn/cn/zh/antibody/product/p16INK4a-Antibody-Polyclonal/PA1-9025	Polyclonal
p53	Abclonal	A5761	WB	1:1000	https://abclonal.com/Datasheet/Antibodies/A5761.pdf	Polyclonal
α-Tubulin	Sigma	T6074	WB	1:5000	https://www.sigmaaldrich.com/US/en/product/sigma/t6074?context=product	Monoclonal
PARP1	Santa Cruz	sc-8007	WB	1:200	https://www.scbt.com/p/parp-1-antibody-f-2	Polyclonal
γ-H2AX	Santa Cruz	sc-517348	IF	1:50	https://www.scbt.com/p/p-histone-h2a-x-antibody-ser-139	Monoclonal

Alexa Fluor 488-conjugated antibodies	Invitrogen	A28175	IF	1:1000	https://www.thermofisher.cn/cn/zh/antibody/product/Goat-anti-Mouse-IgG-H-L-Secondary-Antibody-Recombinant-Polyclonal/A28175	Polyclonal
Rabbit Anti-IgG	Cell signaling	2729	ChIP	10ul	https://www.cellsignal.com/products/primary-antibodies/normal-rabbit-igg/2729	Polyclonal
BrdU mouse mAb	Cell signaling	6813	Cell Proliferation Assay	100ul	https://www.cellsignal.com/products/cellular-assay-kits/brdu-cell-proliferation-assay-kit/6813	Monoclonal
