# Supplementary figures and images for: Post-GWAS functional analysis identifies CUX1 as a regulator of p16INK4a and cellular senescence
Source: Nat Aging. 2022 Feb 17;2(2):140–54. doi: 10.1038/s43587-022-00177-0 (PMC10154215; doi:10.1038/s43587-022-00177-0)

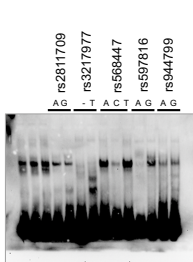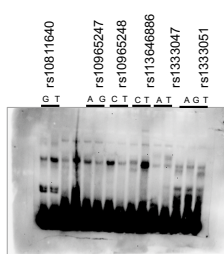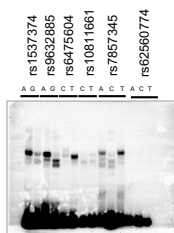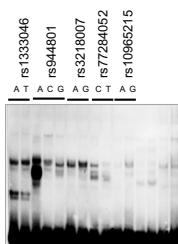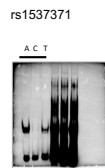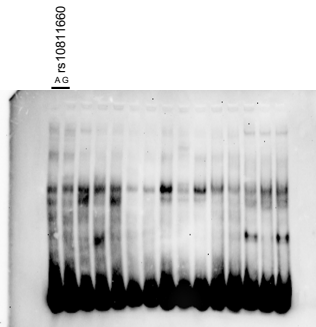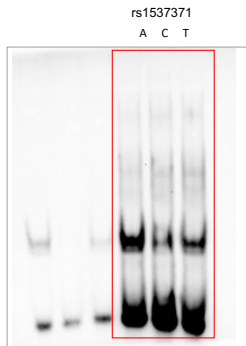

Supplement: Source Data gels and blots. Data Fig. 1 — Unprocessed gels and blots. [file 43587_2022_177_MOESM19_ESM.pdf]

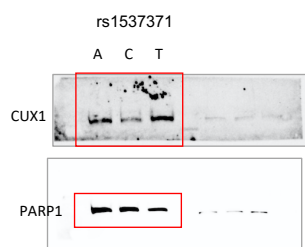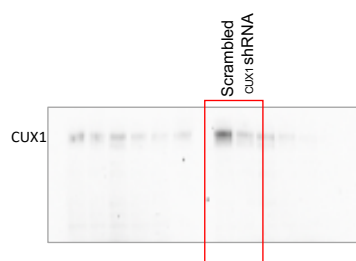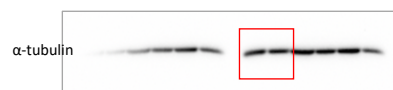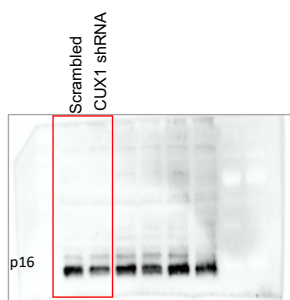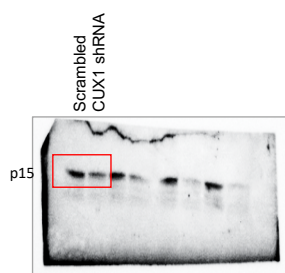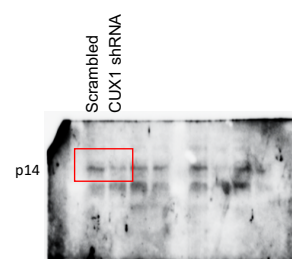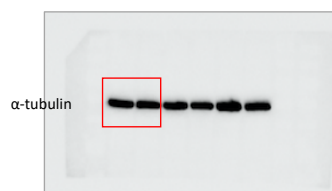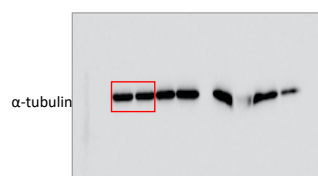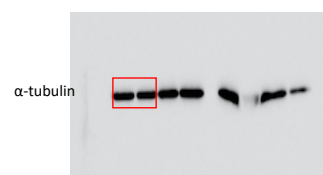

Supplement: Source Data gels and blots. Data Fig. 2 — Unprocessed gels and blots. [file 43587_2022_177_MOESM20_ESM.pdf]

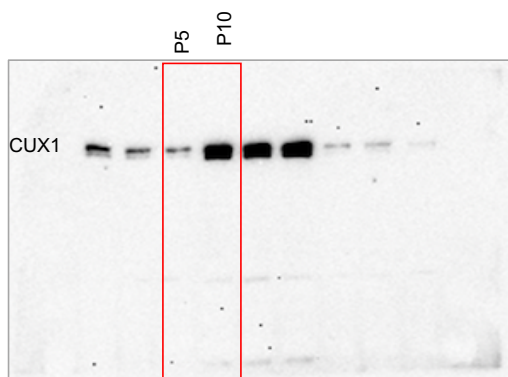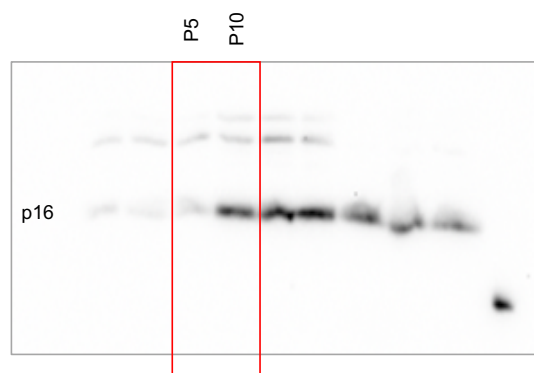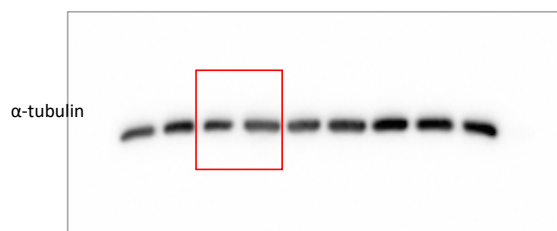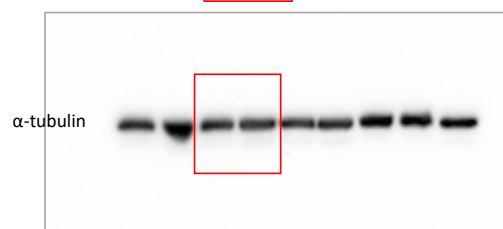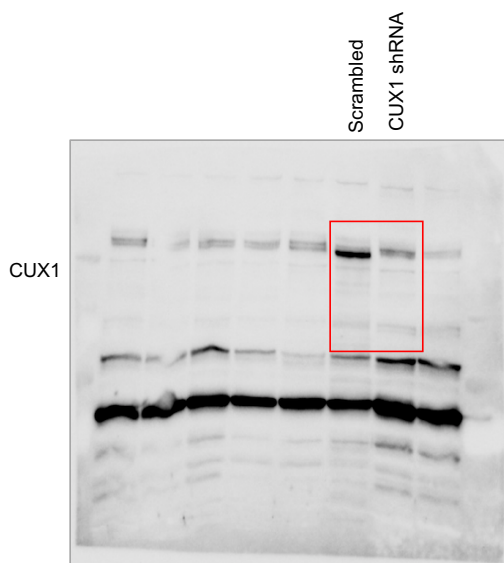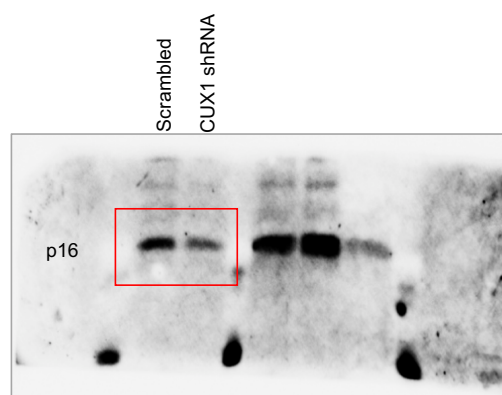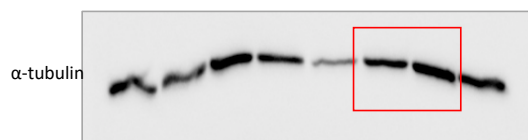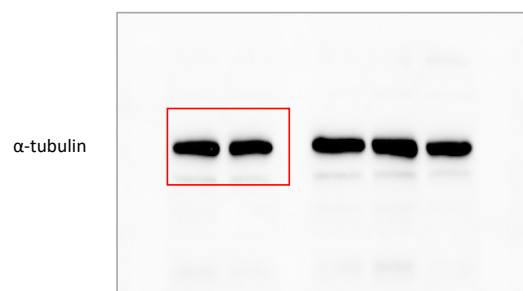

Supplement: Source Data gels and blots. Data Fig. 3 — Unprocessed gels and blots. [file 43587_2022_177_MOESM21_ESM.pdf]

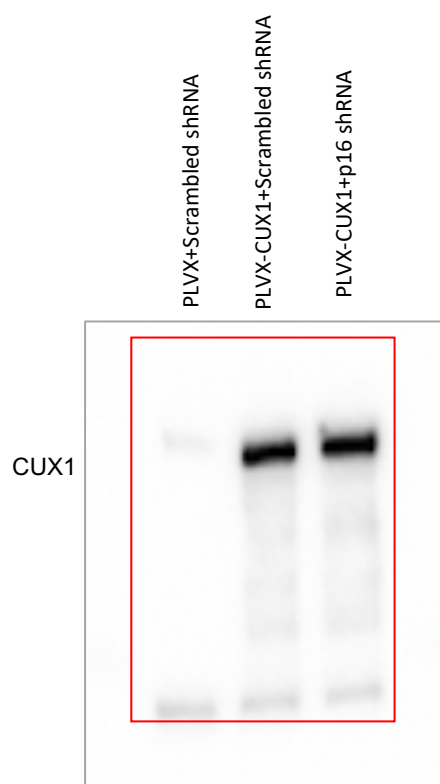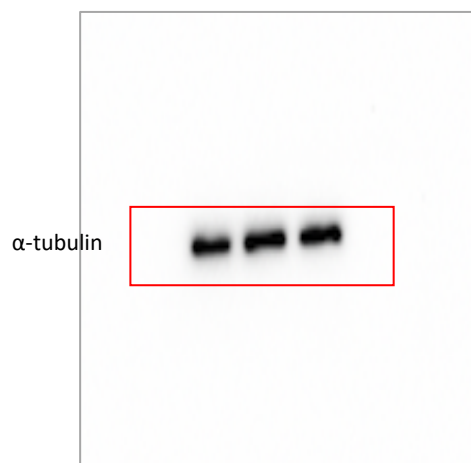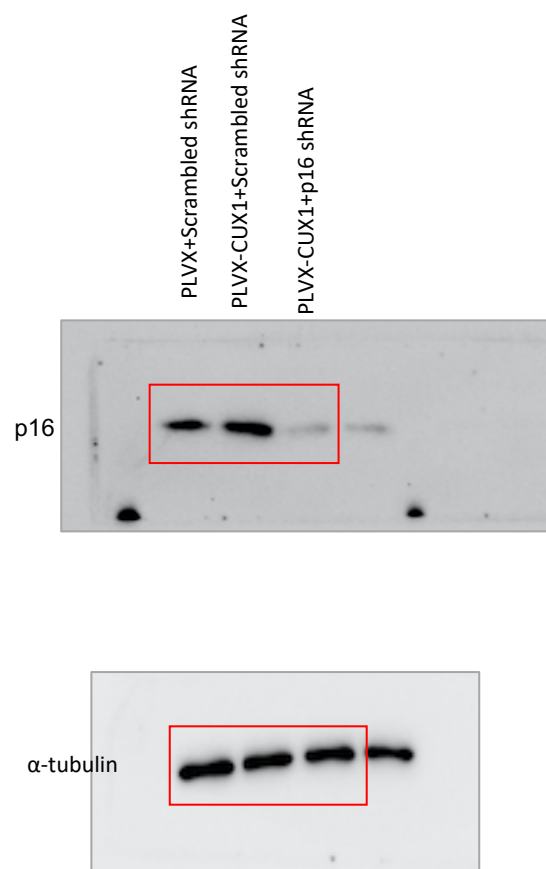

Supplement: Source Data gels and blots. Data Fig. 4 — Unprocessed gels and blots. [file 43587_2022_177_MOESM22_ESM.pdf]

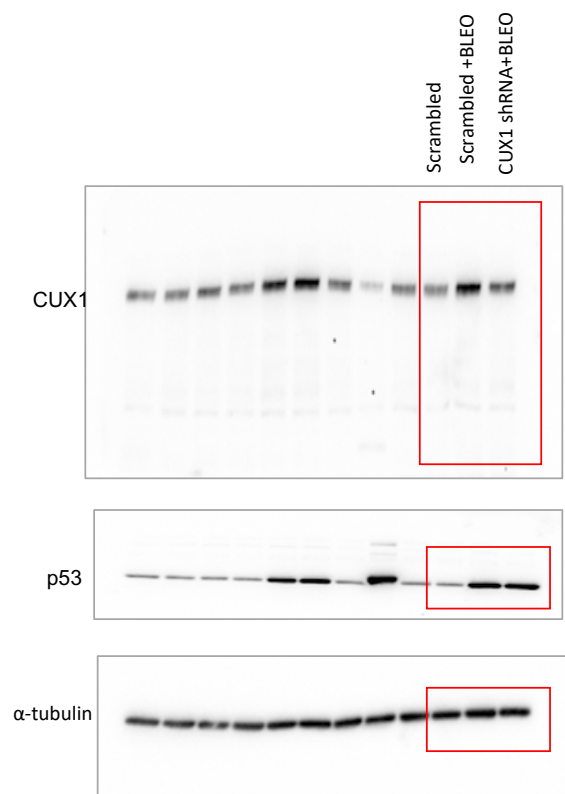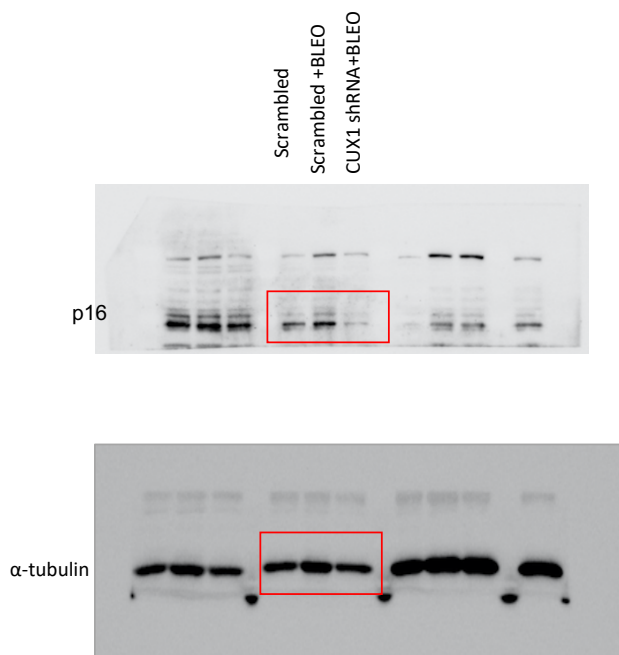

Supplement: Source Data gels and blots. Data Fig. 5 — Unprocessed gels and blots. [file 43587_2022_177_MOESM23_ESM.pdf]

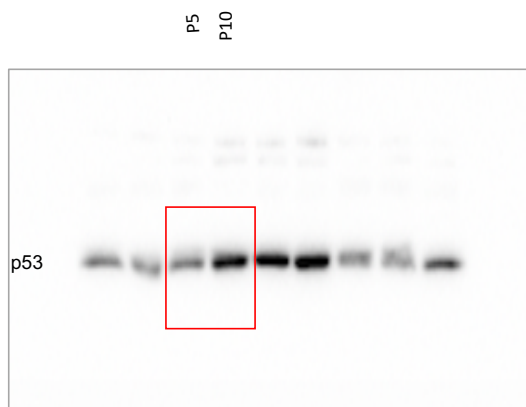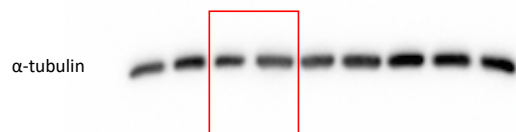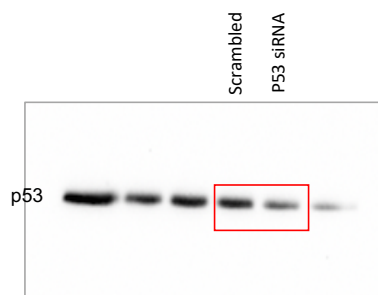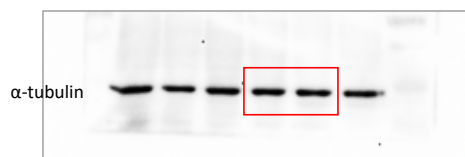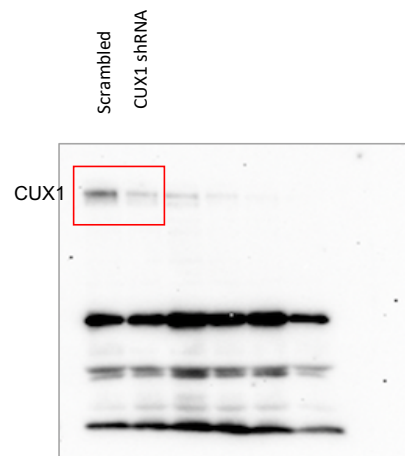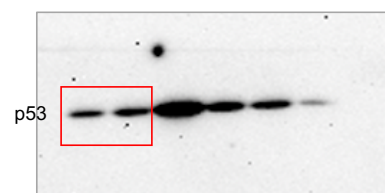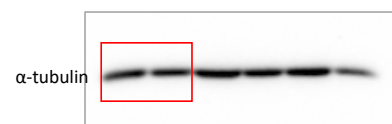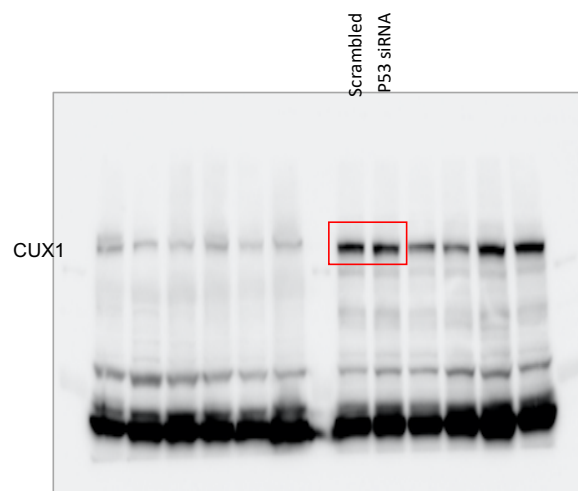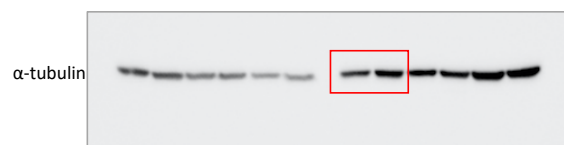

Supplement: Source Data gels and blots. Data Fig. 7 — Unprocessed gels and blots. [file 43587_2022_177_MOESM24_ESM.pdf]

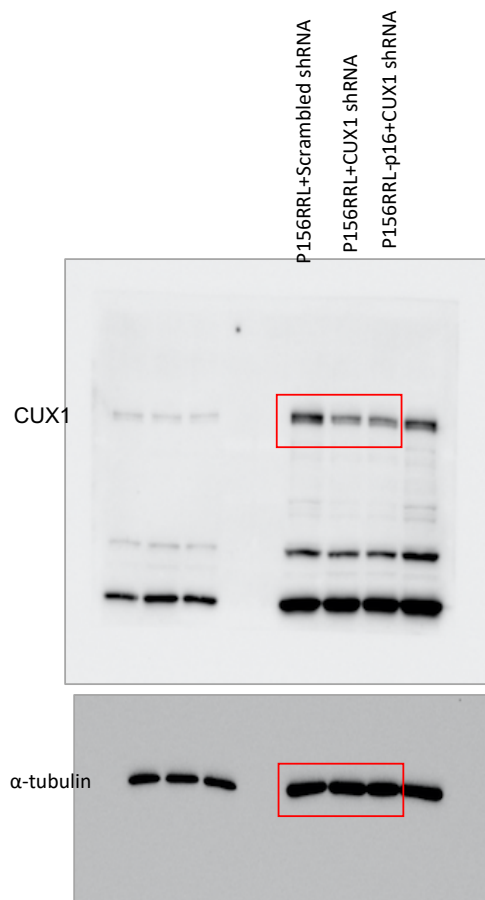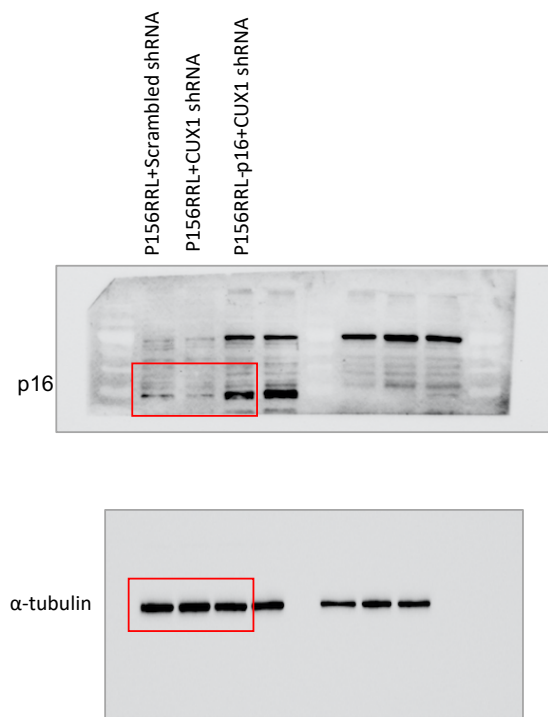

Supplement: Source Data Extended Data gels and blots. Fig. 3 — Unprocessed extended data gels and blots. [file 43587_2022_177_MOESM25_ESM.pdf]

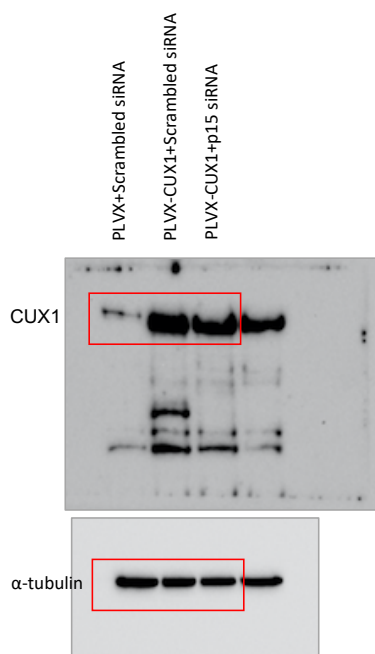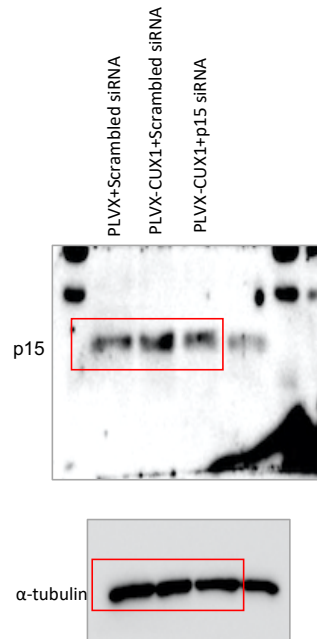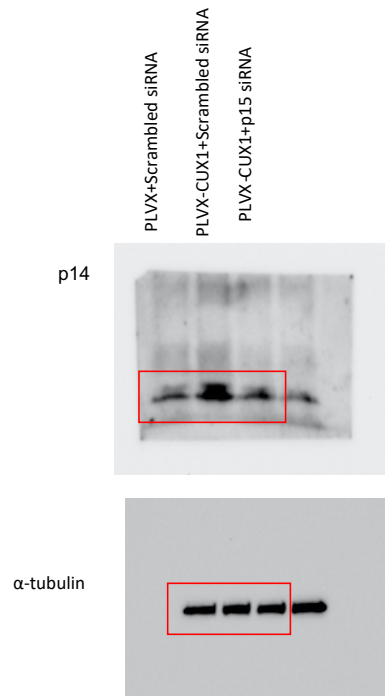

Supplement: Source Data Extended Data gels and blots. Fig. 4 — Unprocessed extended data gels and blots. [file 43587_2022_177_MOESM26_ESM.pdf]

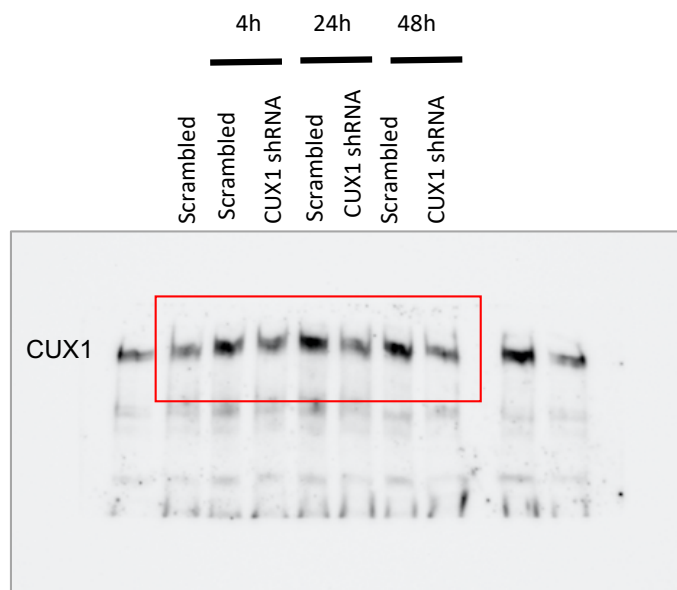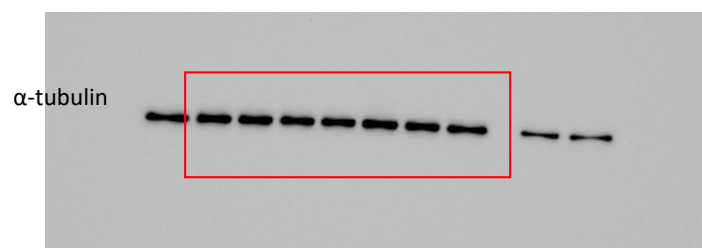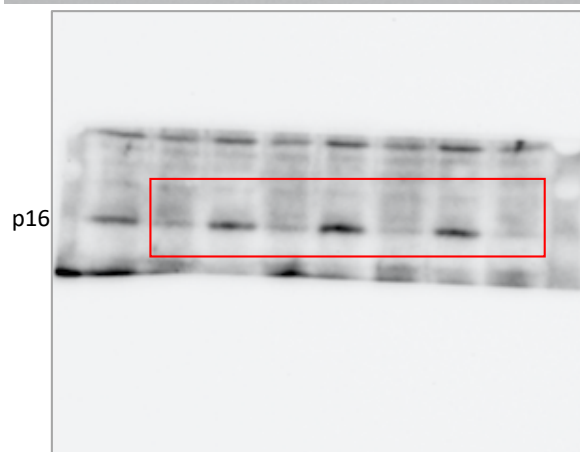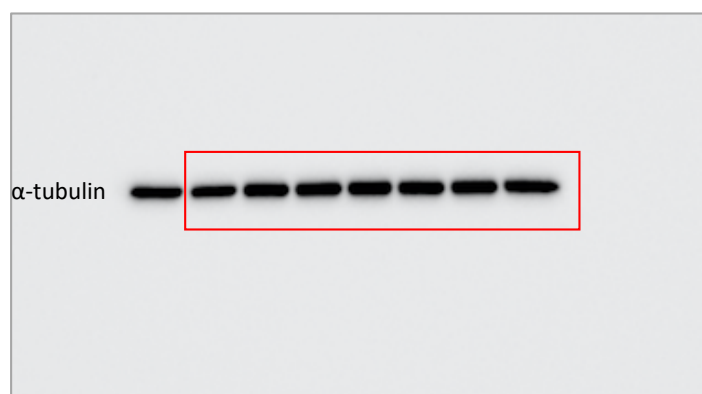

Supplement: Source Data Extended Data gels and blots. Fig. 5 — Unprocessed extended data gels and blots. [file 43587_2022_177_MOESM27_ESM.pdf]

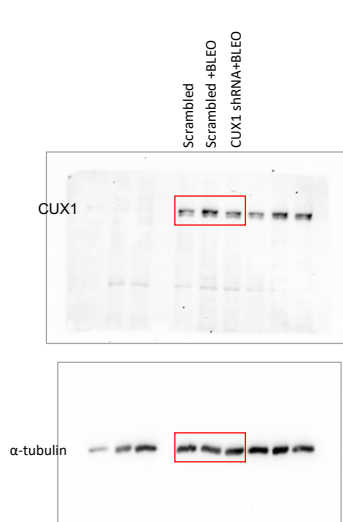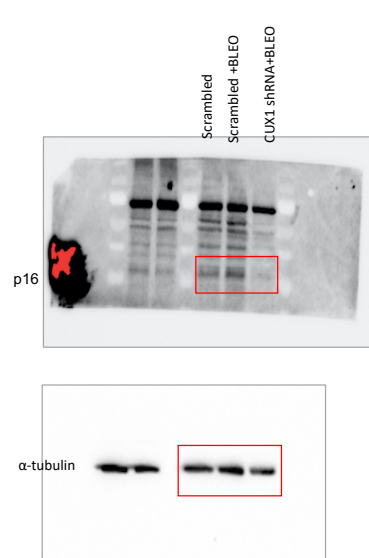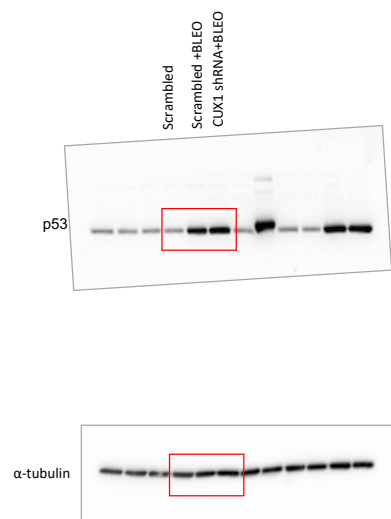

Supplement: Source Data Extended Data gels and blots. Fig. 6 — Unprocessed extended data gels and blots. [file 43587_2022_177_MOESM28_ESM.pdf]
